# NKX6.3 Is a Transcription Factor for Wnt/β-catenin and Rho-GTPase Signaling-Related Genes to Suppress Gastric Cancer Progression

**DOI:** 10.1016/j.ebiom.2016.05.027

**Published:** 2016-05-25

**Authors:** Jung Hwan Yoon, Jung Woo Eun, Won Suk Choi, Olga Kim, Suk Woo Nam, Jung Young Lee, Won Sang Park

**Affiliations:** a Department of Pathology, College of Medicine, The Catholic University of Korea, 505 Banpo-dong, Seocho-gu, Seoul 137-701, South Korea; b Functional RNomics Research Center, College of Medicine, The Catholic University of Korea, 505 Banpo-dong, Seocho-gu, Seoul 137-701, South Korea

**Keywords:** NKX6.3, Wnt/β-catenin, Rho-GTPase, EMT, Gastric cancer

## Abstract

Despite ongoing research and recent progress, the prognosis for patients with advanced gastric cancer remains poor. Wnt/β-catenin and Rho-GTPase signaling pathways are known to play essential roles in malignant transformation and progression of various tumors, including gastric cancer. Here, we identify that NKX6 transcription factor, locus 3 (NKX6.3) binds directly to specific promoter regions of Wnt/β-catenin and Rho-GTPase pathway-related genes, resulting in inhibition of cancer cell migration and invasion. Additionally, we find that the expression level of NKX6.3 is involved in regulation of gastric cancer progression and expression of Wnt/β-catenin and Rho-GTPase pathway-related genes in clinical samples. These results suggest that NKX6.3 prevents EMT and cell migration, implying that NKX6.3 inactivation might be one of the key mechanisms of gastric cancer cell invasion and metastasis.

## Highlights

• NKX6.3 loss is associated with metastasis and poor survival in gastric cancer.
• NKX6.3 transcriptionally inhibits Wnt/β-catenin and Rho-GTPase family induced migration and invasion.
• NKX6.3 inactivation is critical for gastric cancer cell invasion and metastasis.
  Yoon et al. reveal that NKX6.3 prevents EMT and cell migration through transcriptionally modulates the expression of Wnt/β-catenin and Rho-GTPase pathway-related genes, implying that NKX6.3 inactivation might be one of the key mechanisms of gastric cancer cell invasion and metastasis.

## Graphical Abstract

**Figure:**
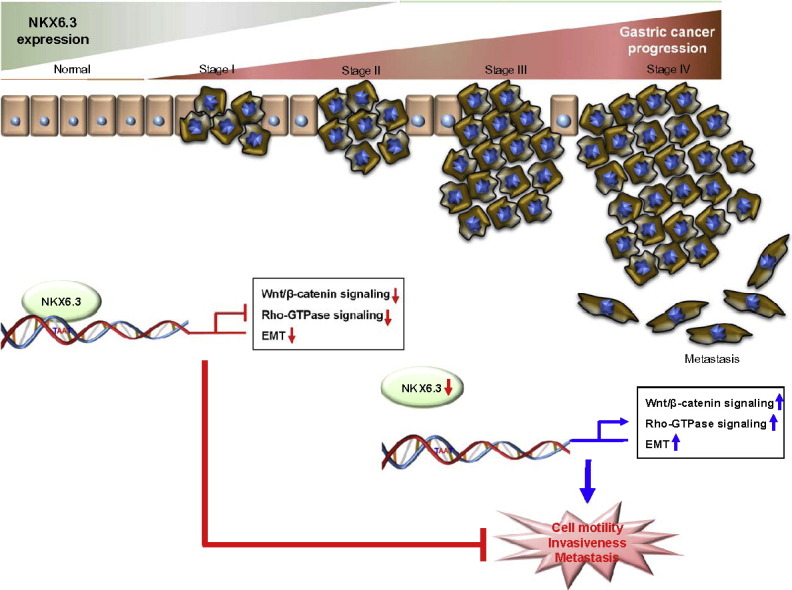

## 1 Introduction

Gastric cancer is one of the leading causes of cancer-related deaths worldwide (Jemal et al., 2011, Jung et al., 2014). The prognosis of patients with gastric cancer remains poor, and in spite of improving surgical resection, combined with efficient adjuvant therapy at the early stages of the disease, ensuing relapse and metastasis often occurs. Moreover, advanced gastric cancers are resistant to traditional therapies and modern treatments are ineffective (Camargo et al., 2014, Xu et al., 2015). Although numerous oncogenes and tumor suppressor genes have been reported to be responsible for the development and progression of gastric cancer (Hu et al., 2012), the molecular mechanisms underlying the gastric cancer cell migration and invasion still remain unclear.

Gastric cancer cells with mesenchymal morphological changes show increased motility and invasiveness due to decreased cell-cell contacts; which are evocative of epithelial-mesenchymal transition (EMT) during embryonic development (Katoh, 2005, Nieto, 2011, Brabletz, 2012, De Craene and Berx, 2013). Since EMT generates cells with invasive properties, able to move into surrounding normal tissues or organs, identification of signals that lead to EMT remains a central challenge in cancer research. A hallmark of EMT is the functional loss of epithelial markers and the up-regulation of mesenchymal markers (Peinado et al., 2007). Notably, abnormal activation of the Wnt/β-catenin signaling pathway leads to β-catenin accumulation in the nucleus and subsequent binding to the T cell factor (TCF) or lymphoid enhancer factor (LEF) transcription factors, thereby inducing the expression of Wnt/β-catenin target genes, which are essential for EMT and metastatic progression of epithelial cancers (Reya and Clevers, 2005, Nguyen et al., 2009, Zhang et al., 2009, Khew-Goodall and Goodall, 2010). Therefore, a better understanding of the molecular mechanisms of Wnt/β-catenin signaling, would provide a crucial insight into how EMT and cancer progression might be regulated.

The NKX transcription factor family is involved in a variety of developmental processes (Alanentalo et al., 2006). Specifically, NKX6.3 transcript is localized in the epithelium of the mouse distal stomach region and acts as a selective regulator of G- and D-cell lineages (Choi et al., 2008). Recently, we have reported that somatic or epigenetic change of the *NKX6.3* gene was not found, whereas decreased DNA copy number of *NKX6.3* and frequent allelic loss (52.2%) at the *NKX6.3* locus was detected in gastric cancer (Yoon et al., 2015). Moreover, we found that NKX6.3 protein is expressed ubiquitously across all gastric glandular epithelial cells in humans, and functions as a key transcriptional regulator of the genes that are involved in cell fate, including growth, differentiation and death (Yoon et al., 2015, Yoon et al., 2016). We predicted that it is likely that NKX6.3 plays an important role in maintaining gastric mucosal integrity and may act as functional tumor suppressor. The physiological relevance of the crosstalk between NKX6.3 and Wnt/β-catenin signaling in cancer has yet to be elucidated.

In this study, we have examined the roles of NKX6.3 and Wnt/β-catenin signaling in the progression of gastric cancer. Our study reveals that NKX6.3 is a critical regulator of the Wnt/β-catenin, Rho-GTPase signaling pathways and gastric cancer progression.

## 2 Materials and Methods

### 2.1 Human Gastric Samples

A total of 65 frozen gastric cancers were obtained from the Chonnam National University Hwasun Hospital, which is supported by the Ministry of Health, Welfare and Family Affairs. Informed consent was provided according to the Declaration of Helsinki. Written informed consent was obtained from all subjects. The study was approved by the Institutional Review Board of The Catholic University of Korea, College of Medicine (MC15SISI0015). There was no evidence of familial cancer in any of the patients.

### 2.2 Immunohistochemistry for NKX6.3

For the immunohistochemical analysis, tissue microarray recipient blocks were constructed containing 157 gastric cancer tissues from formalin-fixed paraffin embedded specimens. Three tissue cores from each cancer (2 mm in diameter) were taken and placed in a new recipient paraffin block using a commercially available microarray instrument (Beecher Instruments, Micro-Array Technologies, Silver Spring, MD, USA), according to established methods (Kononen et al., 1998). One cylinder of normal gastric mucosa adjacent to each tumor was also transferred to the recipient block. 2 μm sections were cut the day before use and stained according to standard protocols.

To maximize immunohistochemistry signal, two strategies were used: antigen retrieval in citrate buffer, and signal amplification with biotinylated tyramide, as previously described (Park et al., 2000). The sections were incubated overnight at 4 °C with NKX6.3 antibodies (1/100; Atlas antibodies, Stockholm, Sweden). Detection was carried out using biotinylated goat anti-rabbit antibodies (Sigma, St. Louis, MO, USA), followed by incubation with a peroxidase-linked avidin-biotin complex. Diaminobenzidine was used as a chromogen, and the slides were counterstained with Mayer's hematoxylin. Staining for NKX6.3 antigen was considered positive when > 30% of the nucleus was stained positively. The results were reviewed independently by two pathologists. For negative controls, primary antibodies were replaced with non-immune serum.

### 2.3 Cell culture and Transfection of NKX6.3, Skp2, c-Myc and Mutant β-catenin^S37A^

AGS and MKN1 gastric cancer cells lines were cultured as described in Supplementary information. Complete NKX6.3, Skp2, c-Myc and *mutant* β-catenin^S37A^-cDNA was cloned into the expression vector pCMV6-Myc-DDK (Origene), pcDNA3.1 (Invitrogen) and pQNCX2 (gift from Dr. Eek Hoon Jho, University of Seoul, Seoul, South Korea). We generated stable NKX6.3 transfectants of AGS and MKN1 cells, AGS^NKX6.3^ and MKN1 ^NKX6.3^, stably expressing human NKX6.3, as well as mock transfectants, AGS^Mock^ and MKN1^Mock^ cells, as described previously and in the Supplemental information (Yoon et al., 2015). Stable expression of NKX6.3 was confirmed in AGSN^KX6.3^ and MKN1^NKX6.3^ cells by western blot analysis.

### 2.4 Cell migration, Invasion Assay and Spheroid Culture

Cell migration, motility, invasion assays and spheroid culture as described in Supplementary information.

### 2.5 Chromatin Immunoprecipitation (ChIP)

ChIP assays were performed using the Thermo Scientific Pierce Agarose ChIP kit (Thermo Scientific Pierce), as described previously and in the Supplemental information (Yoon et al., 2015). DNA amplification was performed by ChIP-qPCR using primers for the promoter described in the Supplemental information.

### 2.6 Cloning of the ChIP Fragments

The immunoprecipitated DNA was cloned as described previously (Yoon et al., 2015). Briefly, the DNA isolated from ChIP was heated at 68 °C for 5 min and then cooled to 37 °C. One to two units of T4 DNA polymerase were added to the DNA and reaction mix containing repair buffer (18 mM ammonium sulfate, 66 mM Tris [pH 8.0], 6.6 mM MgCl_2_, 50 mM β-mercaptoethanol, and 0.5 mM of each nucleotide) and subsequently incubated at 37 °C for 15 min. The reaction was terminated by 1 μl of 0.5 M EDTA for a 50 μl reaction mix. The processed DNA was cloned into pUC118 Hinc II/BAP vector (Takara). Each ligation was transformed into DH-5α competent cells (Clontech). The entire transformation was plated onto ampicillin treated Luria broth agar plates. Randomly picked 120 colonies with inserts were identified by PCR using M13 primers spanning the cloning site in the vector. Inserts > 200 bp were selected for sequencing using a capillary automatic sequencer (3730 DNA Analyzer, Applied Biosystem). The BLAST search of the human genome database at NCBI was performed to locate sequences. The possible genomic binding sequences were identified by pattern matching. Specific sequences were also analyzed using BLAST adjusted to short sequences (Program = blastn, Word size = 7, Expect Value = 100, Filter = disabled). The sequence logo was generated by WebLogo (http://weblogo.berkeley.edu/logo.cgi).

### 2.7 RNA Isolation and Quantitative Reverse Transcriptase (RT)-PCR

Total RNA was extracted from gastric cancer tissues and cell lines following the TRIzol Reagent (Invitrogen) protocol. Two micrograms of total RNA was used in reverse transcription following the Superscript III (Invitrogen) protocol. Quantitative RT PCR was performed on an IQ5 optical system (Bio-rad) using SYBR Green Q-PCR Master Mix (Bio-rad), as described previously and in the Supplemental information (Yoon et al., 2015). Gene expression data were normalized to GAPDH.

### 2.8 Immunoblot and Immunofluorescence (IF)

The effect of NKX6.3 on expression of Wnt/β-catenin, Rho-GTPase signaling and EMT-related proteins including E-cadherin, β-catenin, γ-catenin, Snail, Slug, Vimentin, Zo-1, and ZEB1 was determined in AGS^Mock^, MKN1^Mock^, AGS^NKX6.3^ and MKN1^NKX6.3^ by Western blot, immunofluorescence and confocal microscopy, as described previously and in the Supplemental information (Yoon et al., 2011).

### 2.9 Co-Immunoprecipitation (Co-IP)

AGS^Mock^, MKN1^Mock^, AGS^NKX6.3^ and MKN1^NKX6.3^ cells were washed with PBS and lysed at 4 °C with PBS, pH 7.2 containing 1.0% NP-40, 0.5% sodium deoxycholate, 0.1% SDS, 10 mM NaF, 1.0 mM NaVO4, and 1.0% protease inhibitor cocktail (Sigma, St. Louis, MO, USA) as described previously (Yoon et al., 2015). Briefly, equal protein aliquots (1.0 mg) were immunoprecipitated with 2.0 μg of antibodies to β-catenin (Sigma), E-cadherin (Sigma), and GSK3β (Cell Signaling, Danvers, MA, USA) plus protein A/G-agarose (Santa Cruz Biotechnology, Santa Cruz, CA, USA). Immunoprecipitated proteins were resolved on 12% SDS-polyacrylamide gels and transferred to PVDF membranes (BioRad, Richmond, CA, USA). The membranes were reacted with antibodies against α-, β-, and γ-catenin, E-cadherin, GSK3β, Axin1, and APC each diluted 1:1000. To confirm equivalent protein loading and transfer, the blots were stripped and re-probed for GAPDH (Santa Cruz Biotechnology).

### 2.10 Statistical Analysis

Student's *t*-test was used to analyze the effects of NKX6.3 on cell migration and invasion, ChIP and mRNA expression. Expression of NKX6.3 and clinicopathological factors were analyzed using the Chi-square test. Pearson and linear regression tests were used to analyze the expression of NKX6.3, Wnt/β-catenin and Rho-GTPase signaling-related genes in gastric cancer tissues. Data are expressed as mean ± SEM from at least three independent experiments. A P-value less than 0.05 was considered to be the limit of statistical significance. All experiments were performed in triplicate to verify the reproducibility of findings.

## 3 Results

### 3.1 Altered NKX6.3 Expression Is Strongly Associated with Gastric Cancer Progression

**Fig. 1:**
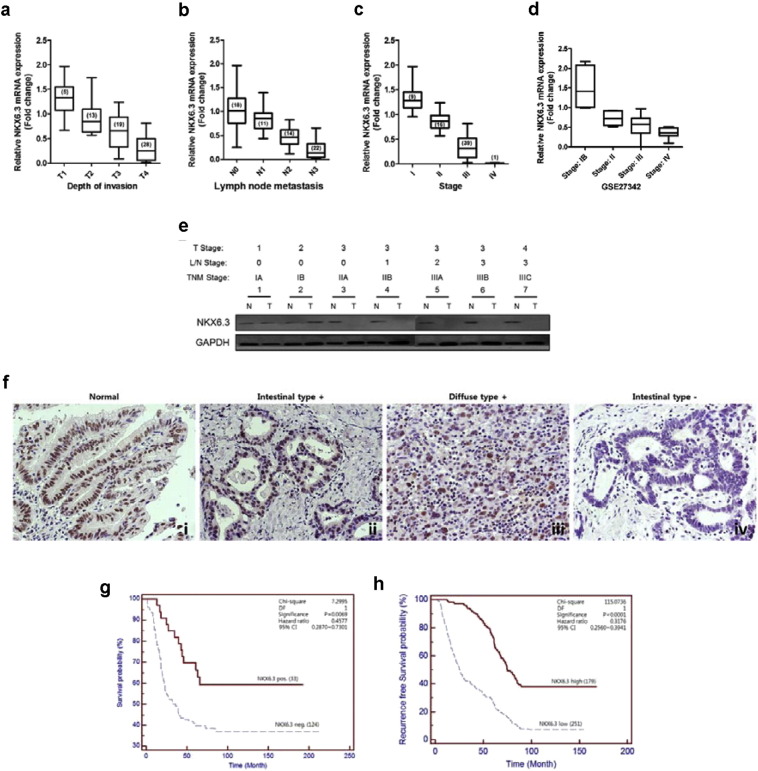
NKX6.3 expression in gastric cancer tissues. (a–c) Expression levels of *NKX6.3* mRNA transcript were significantly lower in gastric cancer samples with; higher T stage (a), lymph node metastasis (b) and TNM stage (c). The results shown are as mean ± SEM of three independent experiments in the same tumors. Numbers in brackets correspond to number of patients. (d) NKX6.3 gene expression was decreased in a large cohort of gastric cancer patients with TNM stage II, III and IV (GSE27342). Numbers in brackets correspond to number of patients. (e) Expression of NKX6.3 protein was lost in gastric tumor tissues of patients of TNM stage II and III. (f) Immunohistochemical analysis of NKX6.3 expression in 157 human gastric tissue specimens. NKX6.3 immunostaning is present throughout the nucleus of gastric mucosa epithelial cells from normal gastric mucosa (i) or intestinal- (ii) and diffuse-type (iii) gastric cancer. In contrast, no NKX6.3 immunoreactivity is present in intestinal-type gastric cancer (iv). (g) Kaplan-Meier plots indicate the overall survival for gastric cancer patients categorized by NKX6.3 expression (n = 33 for positive NKX6.3 group versus n = 124 for negative NKX6.3 group). P value is determined by log-rank test. Association between NKX6.3 expression and clinicopathologic parameters. (h) Kaplan-Meier plot of distant recurrence free survival in a large cohort of 430 gastric cancer patients with low (n = 251) or high (n = 179) *NKX6.3* mRNA expression.

Our previous study showed that NKX6.3 expression was significantly reduced in gastric cancers (Yoon et al., 2015). To investigate whether NKX6.3 contributes to gastric cancer progression, we performed real-time QPCR and western blot in gastric cancer tissues. Expression levels of *NKX6.3* mRNA transcript were significantly lower in gastric cancers with higher T stage (P < 0.05), lymph node metastasis (P < 0.05), and TNM stage (P < 0.05), when compared to gastric cancers with tumor stage T1, without lymph node metastasis (N0) or with TNM stage I (Fig. 1a–c and Table S1). To confirm our results, we recapitulated *NKX6.3* gene expression levels in a large cohort of gastric cancer patients (NCBI GEO database, accession numbers GSE27342) and found that *NKX6.3* expression was also markedly decreased in sporadic gastric cancers with TNM stage II, III, and IV (Fig. 1d).

We also examined the protein expression of NKX6.3 in 65 gastric cancer tissues and corresponding non-tumorous gastric mucosal tissues. Expression of NKX6.3 was absent and/or markedly reduced in tumor tissues of gastric cancer patients with TNM stages II and III (Fig. 1e). When we analyzed the correlation between NKX6.3 protein expression and clinical covariates of these 65 gastric cancer patients, the protein expression of NKX6.3 was not associated with age, gender, site, or histological type (P > 0.05). It was, however, significantly associated with lymph node metastasis (P < 0.0001), depth of invasion (P = 0.0001) and TNM stage (P < 0.0001) in gastric cancer tissues (Table S1). Next, we confirmed the expression and localization of NKX6.3 protein by immunohistochemistry in 157 human gastric cancer tissue specimens. In normal gastric mucosa, we detected NKX6.3 immuno-reactivity in the nucleus of gastric epithelial cells (Fig. 1fi). In gastric cancer tissues, NKX6.3 protein was expressed in neoplastic cells in 33 of 157 (21%) specimens, localized mainly in the nucleus (Fig. 1fii and iii). In addition, its expression was detected in 13 (19.1%) and 20 (22.5%) of 68 intestinal- and 89 diffuse-type gastric cancer, respectively. In contrast, there was no NKX6.3 immunostaining in any of the gastric cancer tissues (Fig. 1fiv). Statistically, there was significant relationship between altered expression of NKX6.3 protein and the clinicopathologic parameters, including depth of invasion and lymph node metastasis (Chi-Square test, P < 0.05) (Table S1). Importantly, Kaplan-Meier analysis revealed that patients that do not express NKX6.3 had on average a 24-month shorter overall survival time, when compared to patients with positive NKX6.3 expression. The median survival time was 49.8 and 73.9 months for patients with negative and positive NKX6.3 expression, respectively (hazard ratio (HR) 0.4577, 95% CI 0.2870-0.7301, P = 0.0069; Fig. 1g). In GEO database (accession number GSE26253), patients with low *NKX6.3* mRNA expression showed significantly worse recurrence free survival (mean of 38.4 versus 82.2 months, hazard ratio (HR) 0.3176, 95% CI 0.2560-0.3941, P < 0.0001; Fig. 1h) than those with high *NKX6.3* mRNA expression.

### 3.2 NKX6.3 Regulates EMT-Related Proteins Implicated in Gastric Cancer Cell Migration and Invasion

**Fig. 2:**
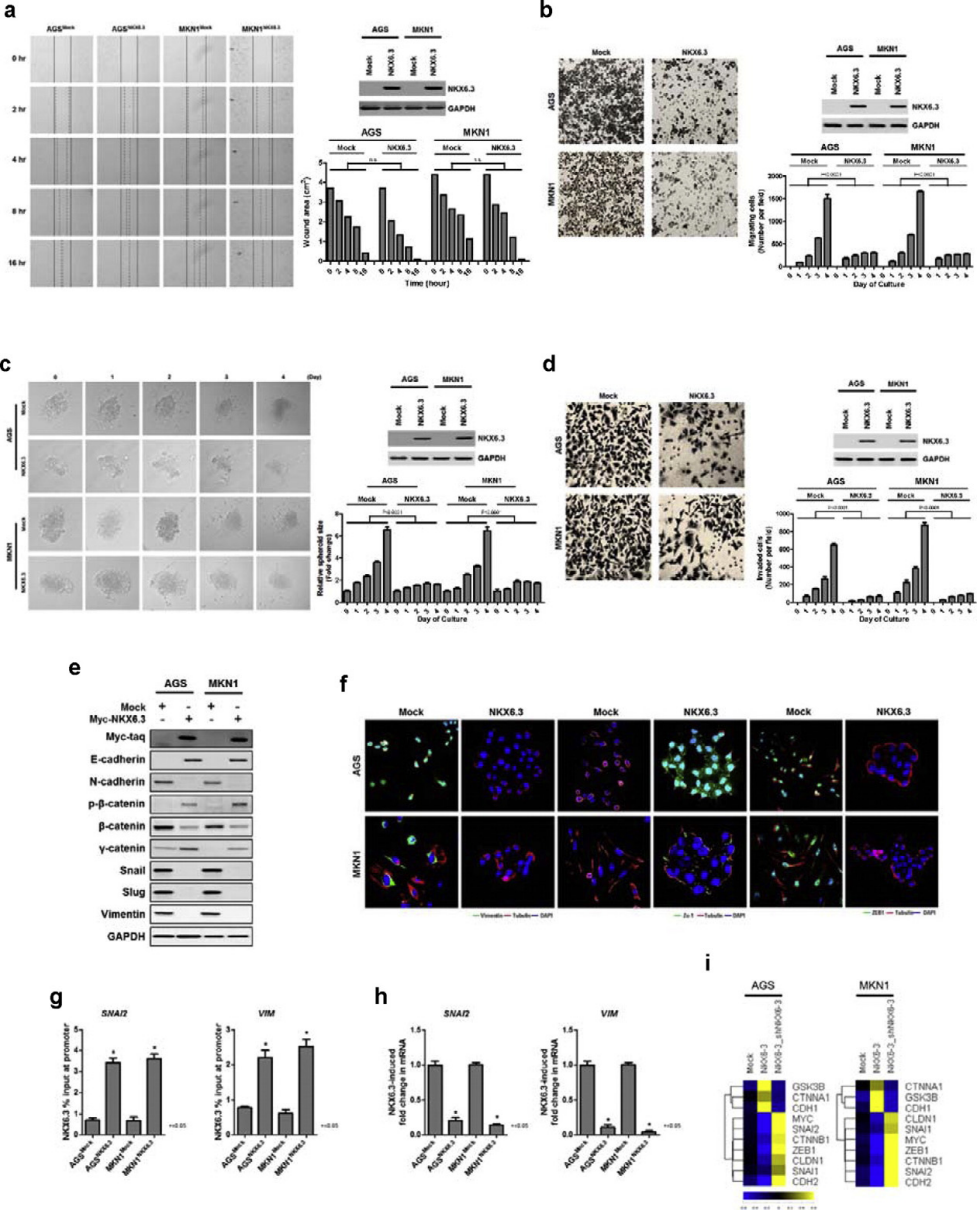
NKX6.3 inhibits gastric cancer cell migration and invasion. (a–c) In vitro wound healing assay, the effect of NKX6.3 cell migration activity was similar to that of mock-stable cells within 16 h (a). NKX6.3 significantly suppressed cell migration at 3 and 4 days after culture in a transwell chemotaxis assay (b). Migration of the spheroidal cancer cells was dramatically reduced in AGS^NKX6.3^ and MKN1^NKX6.3^ at 2, 3 and 4 days after culture (c). The results shown are as mean ± SEM of three independent experiments. (d) Invasiveness of gastric cancer cells was significantly inhibited in AGS^NKX6.3^ and MKN1^NKX6.3^ in a Matrigel-invasion assay. Results represented the mean ± SEM in three independent experiments. (e and f) NKX6.3 induced increased expression of E-cadherin, p-β-catenin and γ-catenin proteins, and reduced expression of N-cadherin, β-catenin, Snail, Slug and Vimentin proteins in western blot assay (e). In an immunofluorescent assay, NKX6.3 enhanced Zo-1 and reduced Vimentin and ZEB-1 protein expression (f). (g) ChIP-qPCR analysis of NKX6.3 binding to the promoter of *SNAI2* and *VIM.* The results represent mean ± SEM from three independent experiments. (h) NKX6.3 suppresses *SNAI2* and *VIM* mRNA expression. The results represent mean ± SEM from three independent experiments. Data were statistically analyzed by Student's *t*-test. (i) Heat-maps show the expression ratios of EMT-related genes modulated by NKX6.3 in AGS and MKN1 cells using quantitative real-time PCR. On the scale bar, yellow indicates up-regulation and blue indicates down-regulation of mRNA compared to mock. Data represent medians of three independent experiments.

To determine whether NKX6.3 contributes to gastric cancer cell migration and invasion, we performed in vitro wound healing, transwell chemotaxis, spheroid-migration and Matrigel invasion assays. Cell migration activity of NKX6.3-stable transfectants at 2, 4, 8 and 16 h after seeding into the wound area, was similar to that of mock-stable cells (Fig. 2a). However, NKX6.3 in AGS^NKX6.3^ and MKN1^NKX6.3^ cells significantly suppressed cell migration at 3 and 4 days after culture in transwell microchemotaxis assays (Fig. 2b). Migration of the spheroidal cancer cells was dramatically reduced in NKX6.3 stable transfectants, at 2, 3, and 4 days after seeding (Fig. 2c).

In a Matrigel assay, invasiveness of gastric cancer cells was significantly inhibited in NKX6.3 stable transfectants, compared to that of mock stable cells at 1, 2, 3, and 4 days after culture (Fig. 2d and S1). Since EMT is a critical process in cell migration and invasion (Scheel and Weinberg, 2012, Puisieux et al., 2014), we further analyzed the function of NKX6.3. As expected, in cancer cells, NKX6.3 expression regulated the expression of EMT-related proteins, such as E-cadherin, Zo-1, N-cadherin, β-catenin, Snail, Slug, Vimentin and ZEB-1 (Fig. 2e and f). In addition, NKX6.3 directly bound to the promoter regions of *SNAI2* and *VIM*, and regulated the EMT-related gene transcripts (Fig. 2g–i and S2). These results suggest that NKX6.3 may inhibit gastric cancer cell migration and invasion by regulating EMT-related protein expression.

### 3.3 NKX6.3 Controls the Wnt/β-Catenin Signaling Pathway

**Fig. 3:**
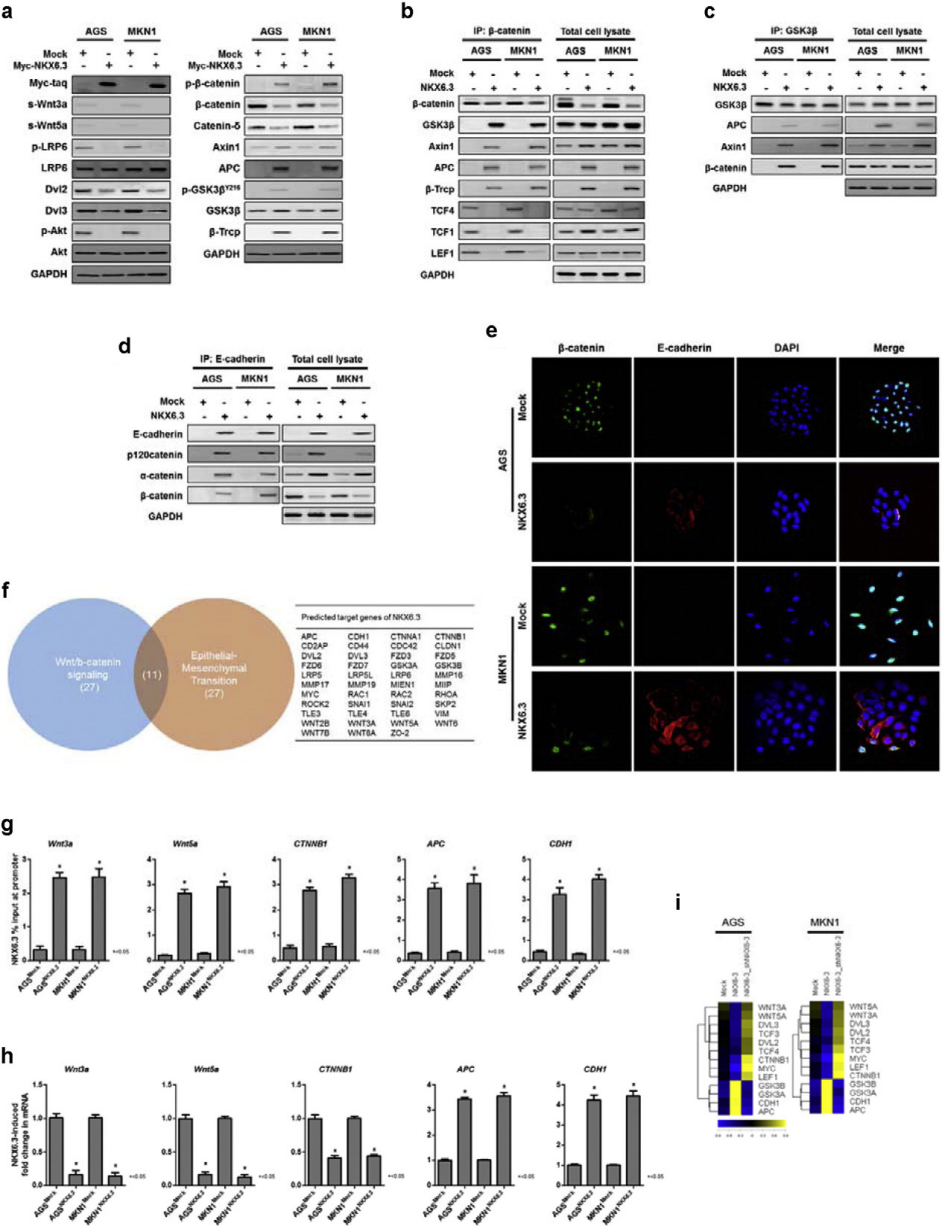
NKX6.3 controls the Wnt/β-catenin signaling pathway. (a) Immunoblots for Wnt/β-catenin signaling pathway-related genes. NKX6.3 inhibits the expression of positive regulators, such as Wnt3a and β-catenin, and induces that of negative regulators, such as APC and β-Trcp. (b and c) NKX6.3 rendered β-catenin to bind to the β-catenin destruction complex of GSK3β, Axin1, APC, and β-Trcp in an immunoprecipitation assay. (d) In immunoprecipitation assay, E-cadherin expression induced by NKX6.3 led to E-cadherin/p120catenin/σ-catenin/β-catenin complex formation. (e) Immunofluorescent staining of β-catenin and E-cadherin. NKX6.3 inhibits nuclear β-catenin expression and induces E-cadherin expression in AGS and MKN1 cells. (f) Venn diagram and a list show that 43 predicted target genes of NKX6.3 are related to the Wnt/β-catenin signaling and EMT. (g) ChIP-qPCR analysis of NKX6.3 binding to the promoters of *Wnt3a*, *Wnt5a*, *CTNNB1*, *APC* and *CDH1.* The results represent mean ± SEM from three independent experiments. (h) NKX6.3 suppresses *Wnt3a*, *Wnt5a*, and *CTNNB1* and induces *APC* and *CDH1* mRNA expression. The results represent mean ± SEM from three independent experiments. Data were statistically analyzed by Student's *t*-test. (i) Heat-maps show the expression ratios of Wnt/β-catenin signaling pathway-related genes modulated by NKX6.3 in AGS and MKN1 cells using quantitative real-time PCR. On the scale bar, yellow indicates u*p*-regulation and blue indicates down-regulation of mRNA compared to mock. Data represent medians of three independent experiments.

The Wnt/β-catenin signaling pathway induces the transcription factor for the EMT-related gene expression and subsequently plays a crucial role in cancer progression and metastasis (Jamora et al., 2003, Reya and Clevers, 2005, Nguyen et al., 2009, Zhang et al., 2009, Khew-Goodall and Goodall, 2010, Qi et al., 2015). Additionally, abnormal Wnt/β-catenin signaling is associated with progression, invasion and metastasis in gastric cancer (Utsunomiya et al., 2000, Zhou et al., 2002, Zhang and Xue, 2008). Thus, we examined if NKX6.3 regulated components of the Wnt/β-catenin signaling pathway. In NKX6.3 stable transfectants, expression of Wnt3a, Wnt5a, p-LRP6, Dvl2, Dvl3, p-Akt, β-catenin and Catenin-δ was markedly decreased, whereas that of p-β-catenin, APC, Axin1, GSK-3β^Y216^, and β-Trcp was increased (Fig. 3a). In immunoprecipitation assays with β-catenin and GSK3β, β-catenin was present in the TCF4/TCF3/LEF1 immunocomplex in AGS^Mock^ and MKN1^Mock^ cells. NKX6.3, however, rendered β-catenin to bind to the β-catenin destruction complex, comprising GSK3β, Axin1, APC, and β-Trcp, in AGS^NKX6.3^ and MKN1^NKX6.3^ cells (Fig. 3b and c). Furthermore, restoration of E-cadherin induced by NKX6.3, led to E-cadherin/p120-catenin/σ-catenin/β-catenin complex formation (Fig. 3d). Immunofluorescent assay also detected loss of nuclear β-catenin expression and restoration of E-cadherin in AGS^NKX6.3^ and MKN1^NKX6.3^ cells (Fig. 3e). We previously reported that NKX6.3 was found to be a transcription factor for various genes (Yoon et al., 2015). As such, we further examined the role of NKX6.3 as a transcription factor for Wnt/β-catenin signaling pathway-related genes by ChIP-cloning and sequencing analysis. As shown in Fig. 3f, 43 genes related to the Wnt/β-catenin signaling and EMT were predicted target genes of NKX6.3. Notably, ChIP analysis confirmed that NKX6.3 binding activity increased in promoter regions of selected Wnt/β-catenin signaling pathway-related genes, such as *Wnt3a*, *Wnt5a*, *CTNNB1*, *APC* and *CDH1* (Fig. 3g). In addition, the mRNA expression levels of *Wnt3a*, *Wnt5a* and *CTNNB1* were decreased, and those of *APC* and *CDH1* were increased in AGS^NKX6.3^ and MKN1^NKX6.3^, when compared to AGS^Mock^ and MKN1^Mock^ cells (Fig. 3h, i and S3). Taken together, these results suggest that NKX6.3 may modulate the expression of EMT-related proteins via transcriptional regulation of the Wnt/β-catenin signaling pathway-related genes.

### 3.4 NKX6.3 Prevents Wnt and Mutant β-Catenin Induced Cancer Cell Migration and Invasion

**Fig. 4:**
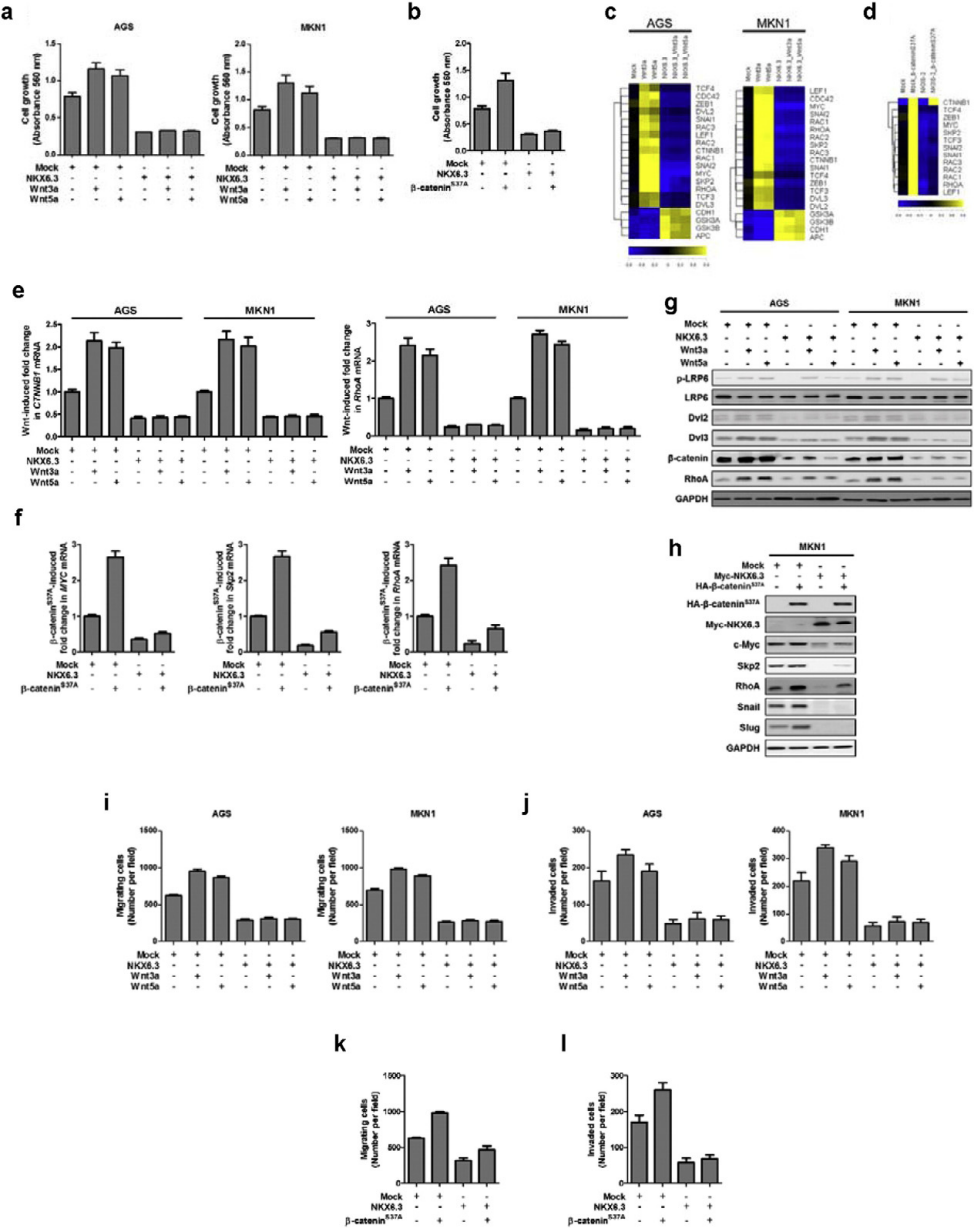
NKX6.3 abrogates Wnt- and mutant β-catenin^S37A^-induced cancer cell migration and invasion. (a) Wnt proteins dramatically increased cell growth, but NKX6.3 failed to promote Wnt-mediated cell growth. The results represent cell growth as mean ± SEM from three independent experiments. Data were statistically analyzed by Student's *t*-test. (b) NKX6.3 inhibits mutant β-catenin^S37A^-mediated cell growth. The results represent cell growth as mean ± SEM from three independent experiments. Data were statistically analyzed by Student's *t*-test. (c) Heat-maps of Wnt-mediated gene expression ratios in AGS and MKN1 cells, examined using quantitative real-time PCR. On the scale bar, yellow indicates up-regulation and blue indicates down-regulation of mRNA compared to mock. The results shown are as mean ± SEM of three independent experiments. (d) Heat-maps of mutant β-catenin^S37A^-mediated gene expression ratios in AGS and MKN1 cells examined using quantitative real-time PCR. On the scale bar, yellow indicates up-regulation and blue indicates down-regulation of mRNA compared to mock. Data are expressed as medians of three independent experiments. (e and g) Wnt proteins induced changes in mRNA (e) and protein (g) expression of Wnt/β-catenin and Rho-GTPase signaling related genes, such as p-LRP6, Dvl2, Dvl3, CTNNB1 and RhoA. NKX6.3 inhibited the Wnt-induced mRNA and protein expression of these genes. The results shown are as mean ± SEM of three independent experiments. (f and h) Real-time PCR (f) and western blot analysis (h) showed that mutant β-catenin^S37A^ induced mRNA and protein expression of c-Myc, Skp2 and RhoA. NKX6.3 significantly reduced the mRNA and protein expression of these genes. Results represented the mean ± SEM of three independent experiments. (i–l) Chemotaxis migration (i and k) and invasion (j and l) assays demonstrated that treatment with Wnt proteins and transfects with mutant β-catenin^S37A^ induced cell migration and invasion. NKX6.3 suppressed Wnt- and mutant β-catenin^S37A^ induced cell migration and invasion. The results are mean ± SEM of three independent experiments.

Wnt proteins induce cancer cell growth, migration and invasion, through the activation of β-catenin and RhoA signaling pathways (Heasman and Ridley, 2008). Additionally, activation of the Wnt/β-catenin signaling pathway is frequently caused by Wnt pathway gene alterations in certain cancers including gastric cancer (Miyoshi et al., 1992, Clements et al., 2002). In cells with *β-catenin* mutations, β-catenin translocates to the nucleus and leads to aberration of the downstream target genes, such as *c-Myc* (He et al., 1998). To investigate whether NKX6.3 is critical for such Wnt- and mutant β-catenin-mediated effects, we examined the impacts of NKX6.3, Wnt proteins (3a and 5a, respectively) and mutant β-catenin^S37A^ on cell growth, migration and invasion. In AGS^Mock^ and MKN1^Mock^ cells, an increase in Wnt proteins and mutant β-catenin^S37A^ dramatically stimulated cell growth, whereas an increase in NKX6.3 failed to promote Wnt- and mutant β-catenin^S37A^-mediated cell growth (Fig. 4a and b). In addition, Wnt proteins induced mRNA and protein expression changes of Wnt/β-catenin and RhoA signaling related genes, such as *CTNNB1*, *Skp2*, *MYC*, *RhoA*, *APC* and *CDH1* (Fig. 4c, e, g and S4). However, NKX6.3 inhibited the Wnt-induced mRNA and protein expression of these genes (Fig. 4c, e, g and S4). Moreover, mutant β-catenin^S37A^ induced mRNA and protein expression of MYC, Skp2 and RhoA, whereas NKX6.3 significantly reduced the expression of these genes (Fig. 4d, f, h and S5). We next examined whether NKX6.3 prevented Wnt and mutant β-catenin^S37A^ induced cell migration and invasion. The chemotaxis migration and invasion assays demonstrated that treatment with Wnt protein and transfection with mutant β-catenin^S37A^ promoted cell migration and invasion, whereas NKX6.3 suppressed the stimulatory effects of Wnt and mutant β-catenin^S37A^ on cell migration and invasion (Fig. 4i–l). These results suggest that NKX6.3 inhibits Wnt and mutant β-catenin^S37A^ induced cell migration and invasion by inactivation of Wnt- and mutant β-catenin^S37A^-mediated β-catenin and RhoA signaling pathways and regulation of β-catenin downstream genes, even in the presence of mutant β-catenin.

### 3.5 NKX6.3 Controls the Expression of Rho-GTPase Family Proteins

**Fig. 5:**
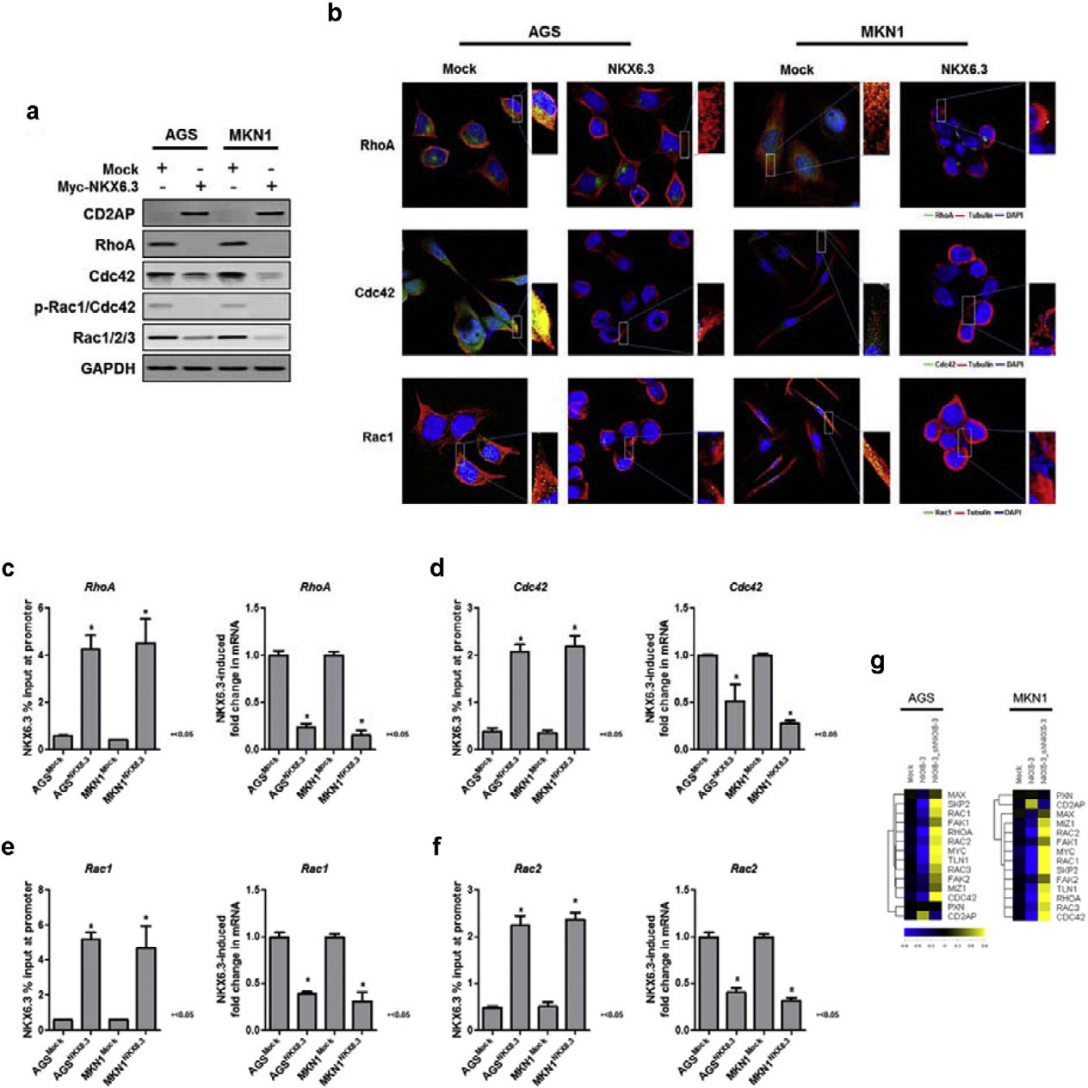
NKX6.3 controls Rho-GTPase family proteins expression. (a) Western blot analysis showed that the expression of all Rho-GTPase family proteins was significantly decreased in AGS^NKX6.3^ and MKN1^NKX6.3^ cells. The expression of CD2AP was increased in NKX6.3 stable AGS^NKX6.3^ and MKN1^NKX6.3^ cells. (b) Immunofluorescence analysis detected strong expression of RhoA, Cdc42, and Rac1 proteins in the cytoplasm of AGS^Mock^ and MKN1^Mock^ cells. NKX6.3 significantly reduced the expression of these proteins in AGS^NKX6.3^ and MKN1^NKX6.3^ cells. (c–f) ChIP-qPCR analysis of NKX6.3 binding to the promoter of *RhoA* (c), *Cdc42* (d), *Rac1* (e) and *Rac2* (f) and their mRNA expression change examined by real-time QPCR. The results are expressed as mean ± SEM of three independent experiments. (g) Heat-maps showing expression ratios of Rho-GTPase signaling pathway-related genes modulated by NKX6.3 in AGS and MKN1 cells using quantitative real-time PCR. On the scale bar, yellow indicates up-regulation and blue indicates down-regulation of mRNA compared to mock. Data are expressed as medians of three independent experiments.

The Rho/ROCK pathway plays an important role in invasion and metastasis in gastric cancer (Matsuoka and Yashiro, 2014). Moreover, the increased expression of RhoA and Rac1 among the Rho-GTPase family proteins is associated with gastric cancer progression (Pan et al., 2004). To determine whether NKX6.3 reduces gastric cancer cell migration through down-regulation of the Rho-GTPase family, we examined the expression of CD2-associated protein (CD2AP), RhoA, Cdc42, p-Rac1/Cdc42 and Rac1/2/3 proteins, in AGS^NKX6.3^ and MKN1^NKX6.3^ cells. Western blot analysis showed that expression of all Rho- GTPase family proteins was significantly decreased, whereas the expression of CD2AP, functioning as a scaffolding protein between cytoskeletal and cell membrane proteins during cell migration (Mustonen et al., 2005), was increased in NKX6.3 stable transfectants, AGS^NKX6.3^ and MKN1^NKX6.3^ cells (Fig. 5a). In immunofluorescence analysis, strong expression of RhoA, Cdc42, and Rac1 proteins was detected in the cytoplasm of AGS^Mock^ and MKN1^Mock^ cells, whereas expression of NKX6.3 significantly reduced the expression of these proteins in AGS^NKX6.3^ and MKN1^NKX6.3^ cells (Fig. 5b). In ChIP and real-time QPCR assays, the binding capacity of NKX6.3 to the promoter regions of *RhoA*, *Cdc42*, *Rac1* and *Rac2* genes was dramatically increased in NKX6.3 stable transfectants and mRNA transcript expression of those genes was significantly decreased in NKX6.3 expressing cells (Fig. 5c–g and S6).

### 3.6 NKX6.3 Functions as a Negative Transcriptional Regulator for RhoA

**Fig. 6:**
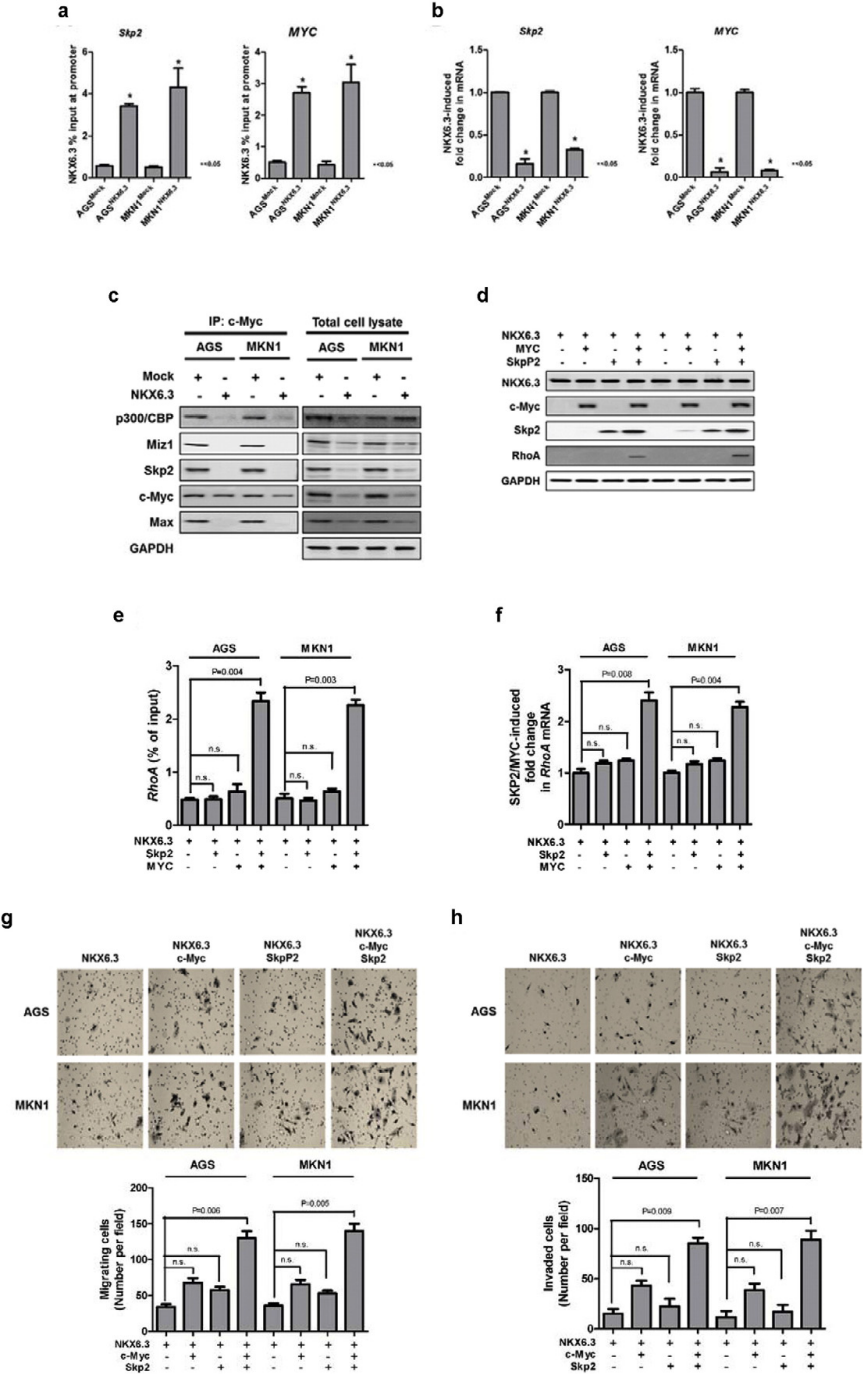
NKX6.3 functions as a negative transcriptional regulator for RhoA. (a) NKX6.3 binding to the promoter of *Skp2* and *c-Myc*. The results are expressed as mean ± SEM of three independent experiments. (b) NKX6.3 significantly reduced *Skp2* and *c-Myc* mRNA expression in real-time QPCR. The results are expressed as mean ± SEM of three independent experiments. Data were statistically analyzed by Student's *t*-test. (c) In AGS^Mock^ and MKN1^Mock^ cells, c-Myc bound to p300/CBP-Skp2-Miz1 and Max proteins, but NKX6.3 markedly inhibited the complex formation in Co-IP assay. (d) Co-transfection of NKX6.3 stable cells with both c-Myc and Skp2. RhoA protein was expressed only in the cells with both c-Myc and Skp2 expression. (e and f) Ectopic expression of both c-Myc and Skp2 significantly enhanced binding activity at the *RhoA* promoter and increased *RhoA* mRNA expression. The results are mean ± SEM of three independent experiments.

The Myc-Skp2-Miz1 transcriptional complex is critical for *RhoA* transcription, cell migration and invasion (Chan et al., 2010). To analyze the effect of NKX6.3 on the c-Myc-Skp2-Miz1 transcription complex, we performed ChIP assays to first address whether NKX6.3 binds to the *Skp2* and *c*-*Myc* promoter regions. As we expected, NKX6.3 bound to the *Skp2* and *c-Myc* promoter (Fig. 6a) and significantly reduced *Skp2* and *c-Myc* mRNA expression in real-time QPCR (Fig. 6b). Next, we examined the effect of NKX6.3 on the formation of the Myc-Skp2-Miz1-p300 complex by co-IP. In AGS^Mock^ and MKN1^Mock^ cells, c-Myc bound to p300/CBP-Skp2-Miz1, and Max proteins, however NKX6.3 markedly inhibited the complex formation (Fig. 6c). In addition, co-transfection with both c-Myc and Skp2 in NKX6.3 stable transfectants significantly increased NKX6.3 binding activity to the promoter region of *RhoA* (Fig. 6d) and induced RhoA protein and mRNA expression (Fig. 6e and f). Furthermore, ectopic expression of both c-Myc and Skp2 in AGS^NKX6.3^ and MKN1^NKX6.3^ cells significantly increased cell migration and invasion (Fig. 6g and h). Taken together, these results suggest that NKX6.3 may suppress gastric cancer cell migration and invasion by inhibiting the Myc-p300/CBP-Skp2-Miz1 transcriptional complex formation required for RhoA expression.

### 3.7 NKX6.3 Is Correlated With Wnt/β-catenin and Rho-GTPase Related Genes in Gastric Cancer Tissues

**Fig. 7:**
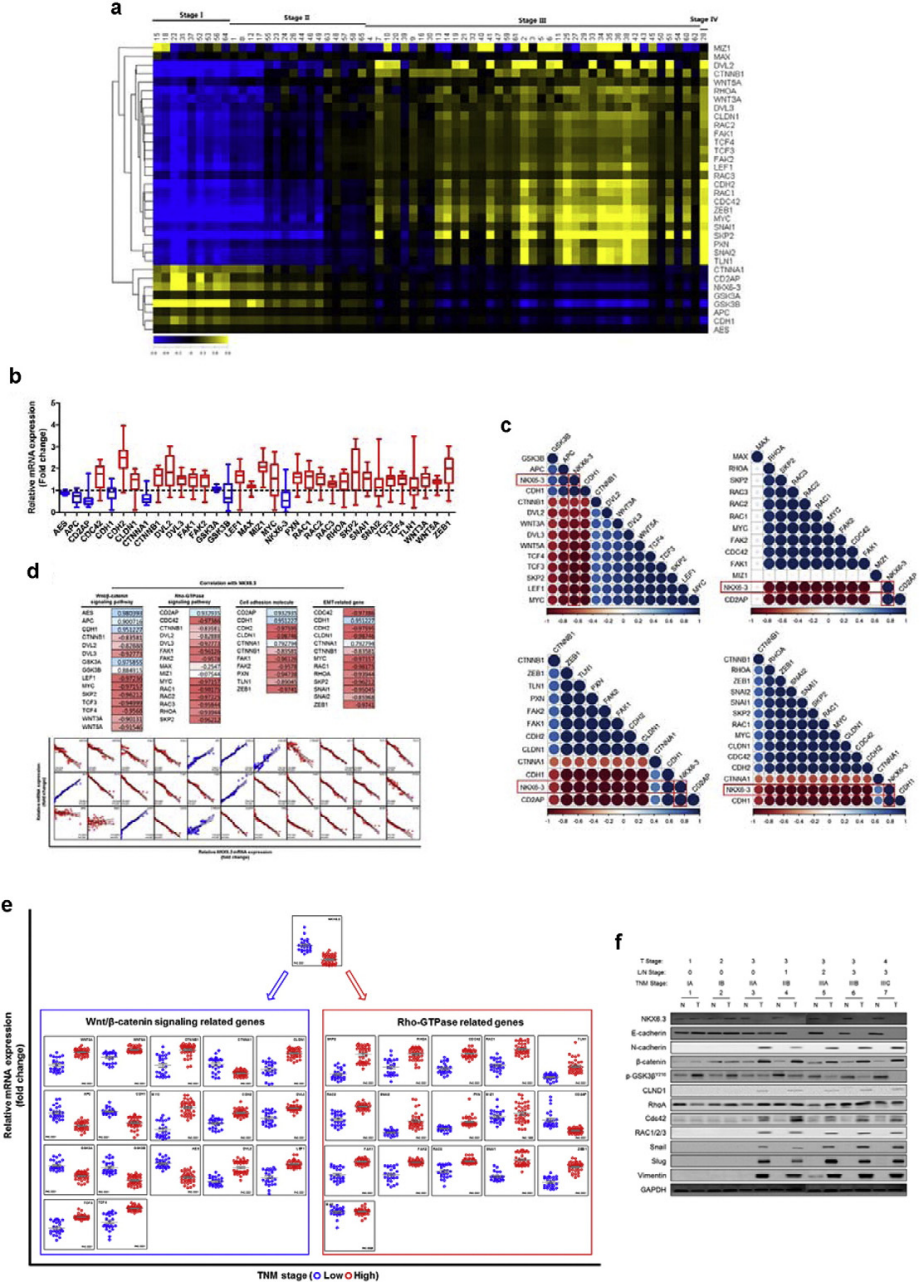
NKX6.3 is negatively correlated with Wnt/β-catenin and Rho-GTPase signaling pathway genes in gastric cancer tissues. (a) Heat-maps demonstrate the expression ratios of NKX6.3, 33 Wnt/β-catenin and Rho-GTPase signaling-related genes, examined using quantitative real-time PCR in 65 gastric cancer tissues. On the scale bar, yellow indicates up-regulation and blue indicates down-regulation of mRNA compared to non-tumorous gastric mucosal tissues. Data are expressed as medians of three independent experiments. (b) Expression of NKX6.3 and 33 Wnt/β-catenin and Rho-GTPase signaling pathway genes in gastric cancer tissues compared to corresponding non-tumorous gastric mucosal tissues. Data are expressed as medians of three independent experiments. (c) Pearson correlation matrices revealed positive (blue) and negative (red) relationships between altered genes. (d) Pearson correlation with NKX6.3, Wnt/β-catenin, Rho-GTPase, cell adhesion and EMT-related genes (blue, positive correlation; red, negative correlation). (e) The effects of NKX6.3 on expression of 33 Wnt/β-catenin and Rho-GTPase signaling pathway genes in gastric cancer tissues with TNM stage (blue dot, stage I, II; red dot, stage III, IV). Data are expressed as medians from three independent experiments. (f) Expression of NKX6.3, E-cadherin, N-cadherin, β-catenin, p-GSK3β^Y216^, CLND1, RhoA, Cdc42, Rac1/2/3, Snail, Slug and Vimentin protein expression in 7 gastric cancer tissues at different TNM stages and corresponding non-tumorous gastric mucosal tissues. Notably, N-cadherin, β-catenin, CLND1, RhoA, Cdc42, Rac1/2/3, Snail, Slug and Vimentin protein expression was increased in tumor tissues of gastric cancer patients with TNM stage II or III. (For interpretation of the references to color in this figure legend, the reader is referred to the web version of this article.)

To further confirm that NKX6.3 reduces gastric cancer cell migration and invasion, we analyzed 33 Wnt/β-catenin, Rho-GTPase signaling-related genes in 65 gastric cancer tissues using real-time QPCR analysis and the gene expression profiles were compared with T stage, lymph node metastasis, and TNM stage. As shown in Fig. 7a, distinctive gene expression profiles were observed according to the T stage, lymph node metastasis, and TNM stage (Fig. 7a; Fig. S7a and b). In addition, the expression of positive and negative regulators of Wnt/β-catenin and Rho-GTPase signaling pathways were up- and down-regulated in gastric cancer tissues, respectively (Fig. 7b). Furthermore, Pearson correlation matrices revealed positive (blue) and negative (red) relationships between altered genes (Fig. 7c). Then we examined whether the expression level of *NKX6.3* mRNA transcript associates with those of Wnt/β-catenin, Rho-GTPase signaling pathways, cell adhesion and EMT-related genes. Pearson correlation values for the positively (blue) associated genes ranged from 0.98 (NKX6.3 versus AES) to 0.07 (NKX6.3 versus MIZ1); and for the inversely (red) associated genes ranged from − 0.25 (NKX6.3 versus MAX) to − 0.99 (NKX6.3 versus CLDN1) (Fig. 7d). As expected, when we compared relative mRNA expression of the above genes between gastric cancer cases with low (I/II) and high (III/IV) TNM stage, the expression of genes that were positively correlated with NKX6.3 was significantly decreased, whereas that of negatively correlated ones was dramatically elevated (Fig. 7e). Next, we determined the expression of NKX6.3, E-cadherin, N-cadherin, β-catenin, p-GSK3β^Y216^, CLND1, RhoA, Cdc42, Rac1/2/3, Snail, Slug and Vimentin proteins in 7 gastric cancer tissues at different TNM stages and compared with expression in non-tumorous gastric mucosal tissues. Notably, expression of CLND1, β-catenin and Vimentin proteins was increased in gastric cancer tissue from patients with TNM stage II or III (Fig. 7f). In contrast, p-GSK3β^Y216^ expression was increased in gastric cancer tissues from patients with TNM stage I, but reduced in gastric cancer tissues from patients with TNM stage II or III. Finally, we conclude that NKX6.3 may inhibit the progression of gastric cancer by down-regulating β-catenin signaling pathway and Rho-GTPase family.

## 4 Discussion

Disruption of gastric epithelial homeostasis may result in abnormal cell proliferation, invasion, and metastasis, which are recognized as cancer hallmarks. During tumor progression, subsequent invasiveness is thought to herald the onset of the last stage of a multi-step process that eventually leads to metastatic dissemination with life-threatening consequences (Thiery, 2002).

EMT has been shown to occur in wound healing, organ fibrosis and the initiation of cancer metastasis (Kalluri and Weinberg, 2009). EMT in cancer cells of epithelial origin is a crucial step that precedes the induction of motility and invasive potential during metastatic progression (Chaffer and Weinberg, 2011, Dave et al., 2012). Numerous studies revealed signaling pathways, such as TGF-β, NF-κB, and Wnt/β-catenin that are involved in regulation of EMT (Lee et al., 2006, Chaudhury et al., 2010, Howard et al., 2011, Lamouille et al., 2014). It was reported that the Wnt signaling pathway inhibits GSK3β and stabilizes β-catenin, which translocates to the nucleus to engage LEF and TCF transcription factors, resulting in induction of a gene expression program that promotes EMT (Lamouille et al., 2014). Aberrant Wnt signaling caused by abnormal expression of Wnts, and *β-catenin* mutations, is associated with the development and progression of several malignancies, including gastric cancer (Kurayoshi et al., 2006, El Wakil and Lalli, 2011).

Recently, we found that the NKX6.3 protein functions as a key transcriptional regulator, controlling cell growth, differentiation and death, and inhibits gastric tumorigenesis (Yoon et al., 2015, Yoon et al., 2016). In this study, we have investigated whether NKX6.3 is implicated in gastric cancer progression mechanisms and acts as functional tumor suppressor through affecting Wnt/β-catenin and Rho-GTPase signaling pathways. Here, we show that NKX6.3 expression level is negatively associated with tumor stage, lymph node metastasis, and TNM stage in 65 gastric cancer tissues (Fig. 1). In vitro, NKX6.3 significantly inhibits migration and invasion of gastric cancer cells (Fig. 2). Interestingly, NKX6.3 induced E-cadherin and γ-catenin expression with a concomitant decline in mesenchymal marker expression, including N-cadherin, Snail, Slug, and Vimentin, suggesting that NKX6.3 is able to inhibit EMT of gastric cancer cells, not only by inducing epithelial differentiation, but also by suppressing mesenchymal phenotype (Fig. 2). Moreover, NKX6.3 directly bound to promoter regions of *SNAI2* and *VIM* and reduced mRNA transcripts of the *SNAI2* and *VIM* genes, which are important for behavioral changes related to the adhesion and migration properties for local tumor invasion (Fig. 2). In addition, NKX6.3 induced β-catenin binding to the β-catenin destruction complex (Stamos and Weis, 2013), including GSK3β, Axin1, APC, and β-Trcp, in NKX6.3 stable gastric cancer cells. The resultant re-expression of E-cadherin promoted by NKX6.3 led to E-cadherin/p120-catenin/α-catenin/β-catenin complex formation and facilitated the degradation of cytoplasmic β-catenin (Fig. 3). We further identified a repertoire of NKX6.3 candidate target genes including EMT-related genes and those involved in the regulation of the Wnt/β-catenin signaling pathway (Fig. 3f). NKX6.3 significantly inhibited Wnt- and mutant β-catenin-induced cell growth, migration and invasion by suppressing the Wnt/β-catenin downstream target genes, including *c-Myc* and *RhoA* (Fig. 4).

The Ras homologous (Rho)-GTPase family proteins, including RhoA, Cdc42, and Rac1, are known to play a key role in cancer development and progression mechanisms, including cell transformation, proliferation, migration, invasion and metastasis by regulating the actin cytoskeleton and cell-cell adhesion (Mitchison and Cramer, 1996, Lauffenburger and Horwitz, 1996, Olson et al., 1998, Ridley, 2000, Fukata and Kaibuchi, 2001). Notably, Skp2 induces RhoA transcription, cell migration and invasion by recruiting p300 and Miz1 to the c-Myc complex (Chan et al., 2010).

To elucidate the molecular mechanism by which NKX6.3 inhibits gastric cancer cell migration, we investigated the role of NKX6.3 in Rho-GTPase family protein expressions. Expectedly, NKX6.3 significantly decreased the protein expression of RhoA, Cdc42, p-Rac1/Cdc42, and Rac1/2/3 in gastric cancer cells (Fig. 5). Furthermore, we found that NKX6.3 functions as a negative transcriptional regulator of *RhoA*, *Cdc42*, *Rac1*, *Rac2*, *Skp2* and *c-Myc* genes and completely inhibits c-Myc-p300/CBP-Skp2-Miz1 and Max complex formation, thereby preventing gastric cancer cell migration and invasion (Fig. 6).

Finally, the analysis of 33 Wnt/β-catenin and Rho-GTPase signaling-related genes in 65 gastric cancer tissues showed distinctive gene expression profiles according to the tumor stage, lymph node metastasis, and TNM stage. NKX6.3 expression was positively correlated with expression of Wnt/β-catenin and Rho-GTPase signaling inhibitors, such as APC and GSK3β, and inversely correlated with expression of Wnt/β-catenin and Rho-GTPase signaling pathway activators, such as Wnt3a, Wnt5a, CTNNB1, RhoA and Snail (Fig. 7), confirming that NKX6.3 acts as an inhibitor for Wnt/β-catenin and Rho-GTPase signaling pathways.

Our results support the studies in which Wnt/β-catenin and Rho-GTPase signaling pathways stimulated gastric cancer malignant transformation and progression. In summary, our findings offer evidence for the functional importance of NKX6.3 in inhibition of EMT and cancer cell migration through suppressing Wnt/β-catenin and Rho-GTPase signaling pathways and the NKX6.3 inactivation could be one of the key mechanisms of gastric cancer cell invasion and metastasis.

## Conflict of Interest Statement

We declare no financial or other relationships that may lead to a conflict of interest in this study.

## Author Contributions

Conceptualization, J.H·Y. and W.S·P.; Methodology, J.H.Y. and W.S.P.; Investigation, J.H.Y.; Formal analysis, J.H.Y., J.W.E., O.K., and W.S.C.; Writing-Original Draft, J.H.Y. and W.S.P.; Writing-Review & Editing, S.W.N. and J.Y.L.; Funding Acquisition, J.H.Y. and W.S.P.; Supervision, W.S.P.

## Appendix A Supplementary data

Supplementary material. Image 2
