# Supplementary material for: NKX6.3 Is a Transcription Factor for Wnt/β-catenin and Rho-GTPase Signaling-Related Genes to Suppress Gastric Cancer Progression
Source: eBioMedicine. 2016 May 25;9:97–109. doi: 10.1016/j.ebiom.2016.05.027 (PMC4972521; doi:10.1016/j.ebiom.2016.05.027)
Supplement: Supplementary file 1 — Supplementary material. [file mmc1.docx]

**Supplemetal Information**

**NKX6.3 is a transcription factor for Wnt/β-catenin and Rho-GTPase signaling-related genes to suppress gastric cancer progression**

Jung Hwan Yoon, Jung Woo Eun, Won Suk Choi, Olga Kim, Suk Woo Nam, Jung Young Lee, Won Sang Park

Supplemental Data

**
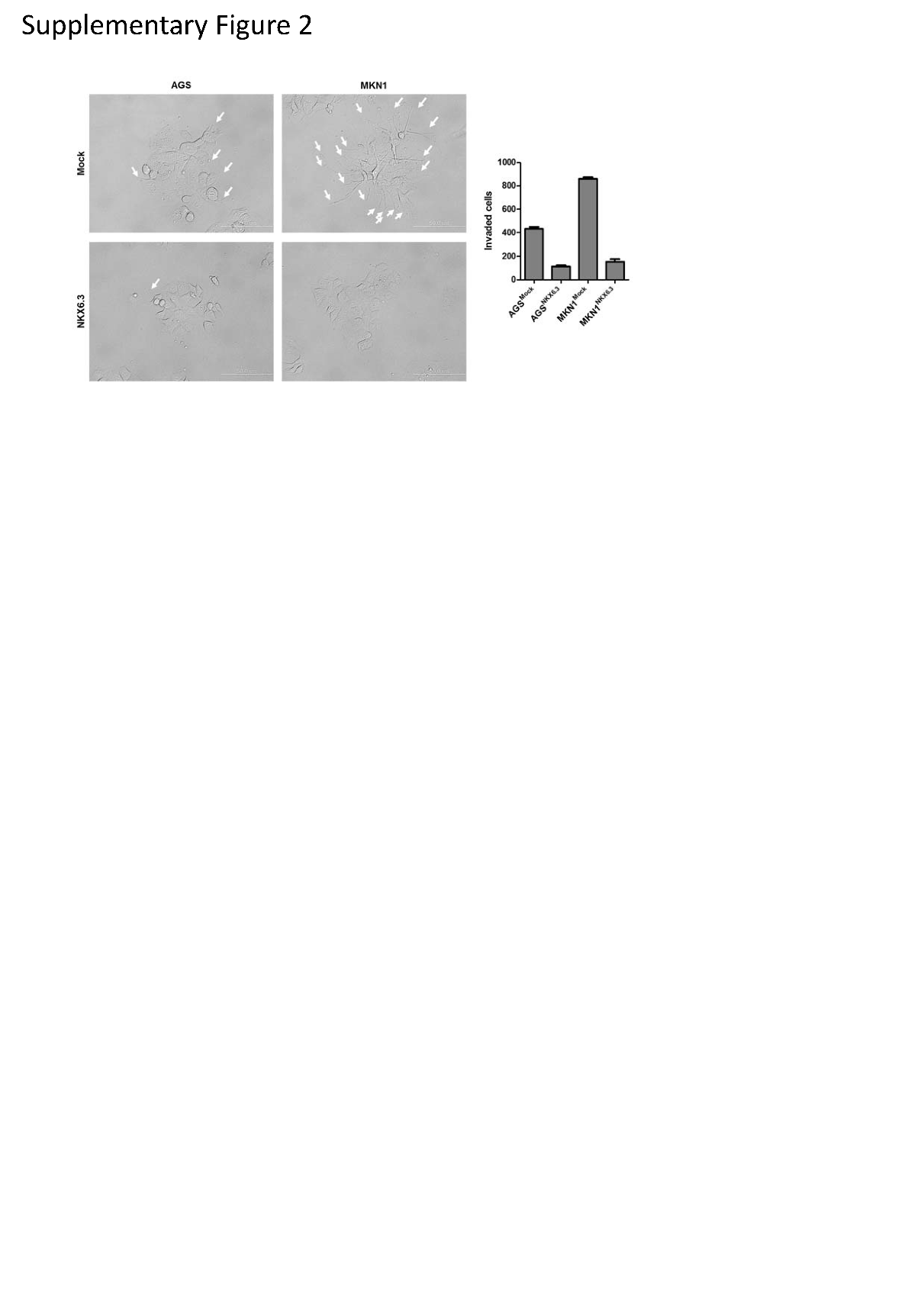
**

**Figure S1 (Related to Fig. 2a-d). Cell morphological changes and invasiveness were inhibited by NKX6.3 in AGS and MKN1 cells.**

Spindle-shaped AGS and MKN1 cells were converted to circular-shaped epithelial cells and invasiveness of gastric cancer cells was significantly suppressed by NKX6.3 expression in a Matrigel-invasion assay.

**
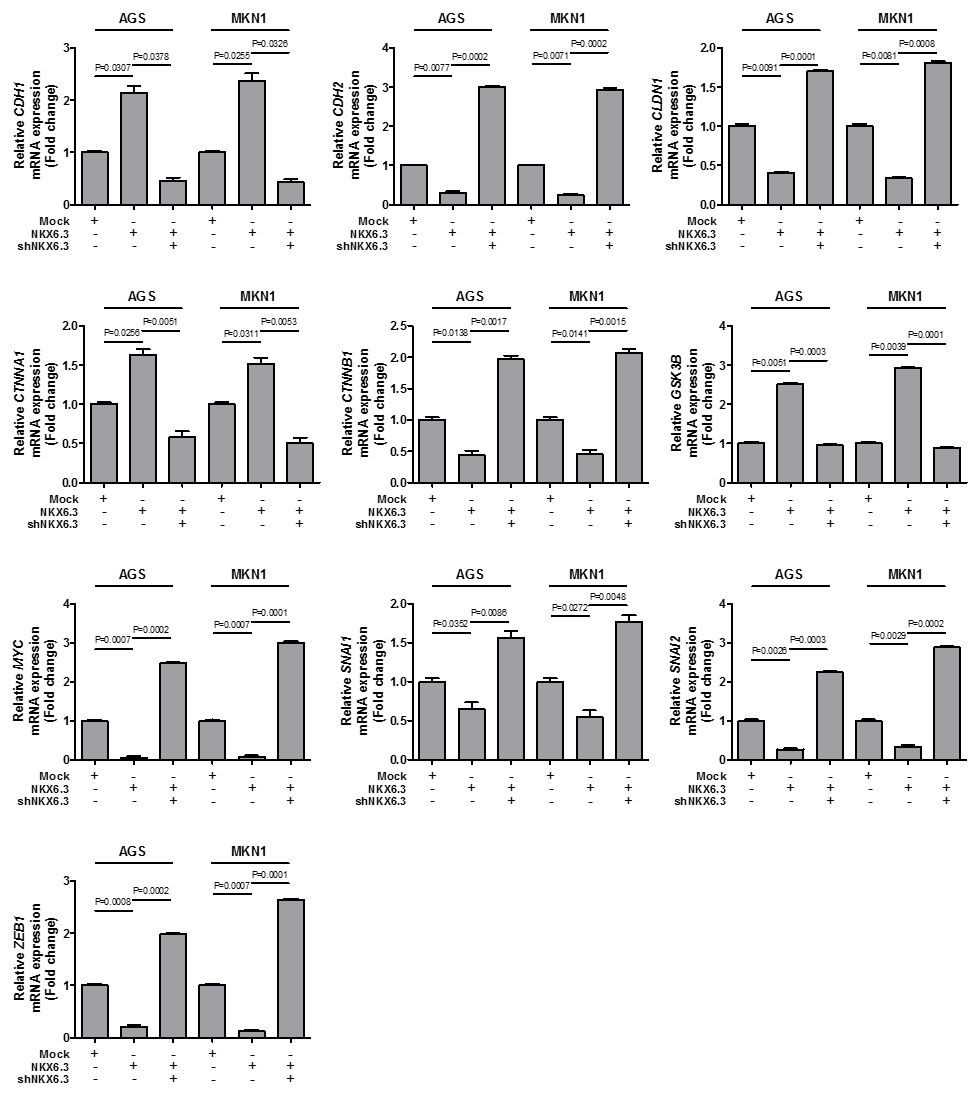
**

**Figure S2 (Related to Fig. 2i). mRNA expression of EMT-related genes modulated by NKX6.3.**

Real-time QPCR for EMT-related gene expressions. NKX6.3 suppresses the mRNA expression of *CDH2*, *CLDN1*, *CTNNB1*, *MYC*, *SNAI1*, *SNAI2* and *ZEB1*, and induces that of *CDH1*, *CTNNA1* and *GSK3B*. However, mRNA expression of these genes are recovered after silencing of NKX6.3 in AGS and MKN1 cells. The results are expressed as mean±SEM of three independent experiments. Data were statistically analyzed by Student's *t*-test.

**
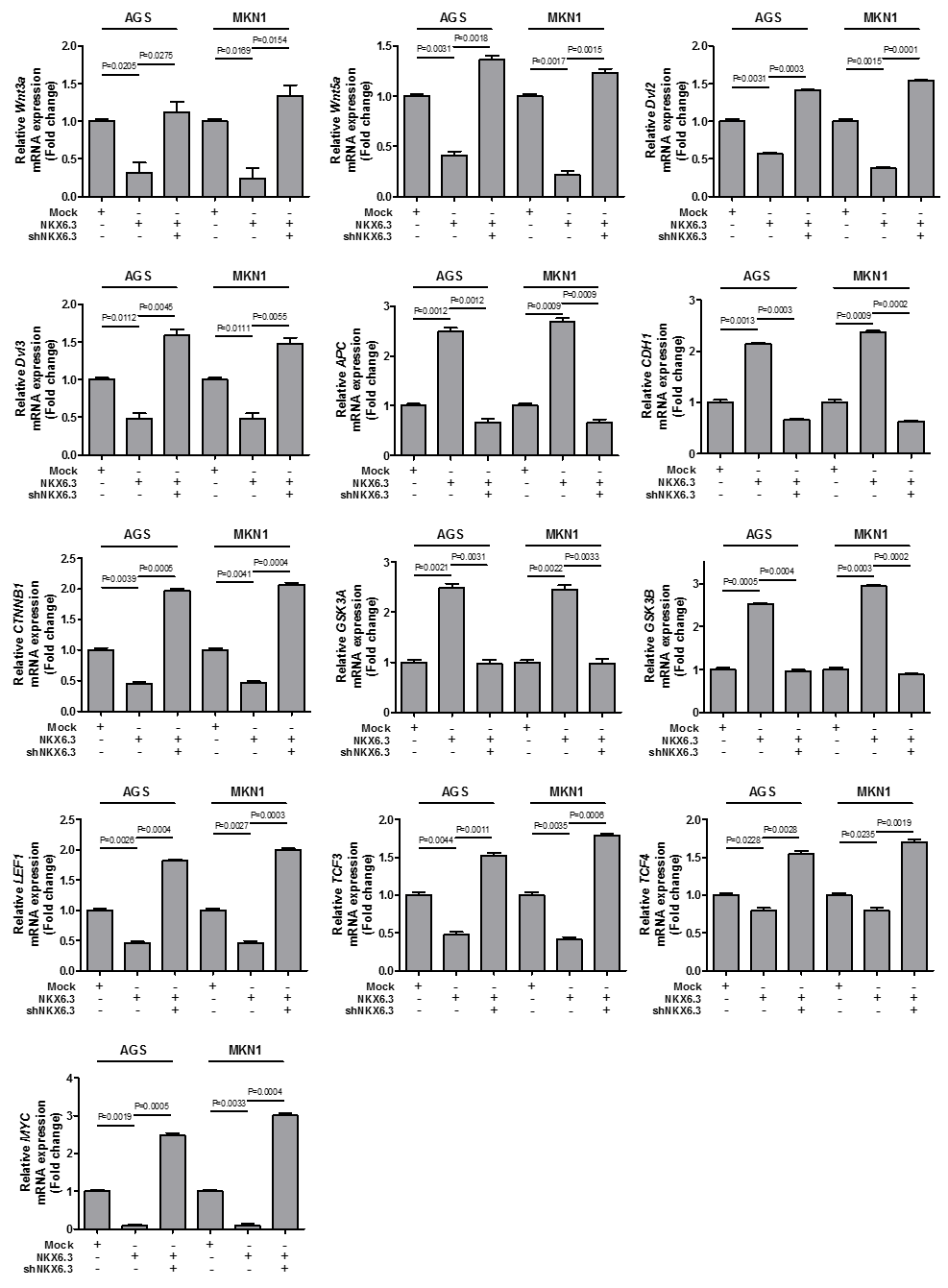
**

**Figure S3 (Related to Fig. 3i). mRNA expression of Wnt/β-catenin signaling pathway-related genes modulated by NKX6.3.**

Real-time QPCR for mRNA expression of Wnt/β-catenin signaling pathway-related genes. NKX6.3 inhibits the mRNA expression of positive regulators, such as *Wnt3a, Wnt5a, Dvl2, Dvl3, CTNNB1* and *LEF1*, and induces that of negative regulators, such as *APC, CDH1, GSK3A* and *GSK3B*. In contrast, mRNA expression of these genes are recovered after NKX6.3 silencing in AGS and MKN1 cells. The results are expressed as mean±SEM of three independent experiments. Data were statistically analyzed by Student's *t*-test.

**
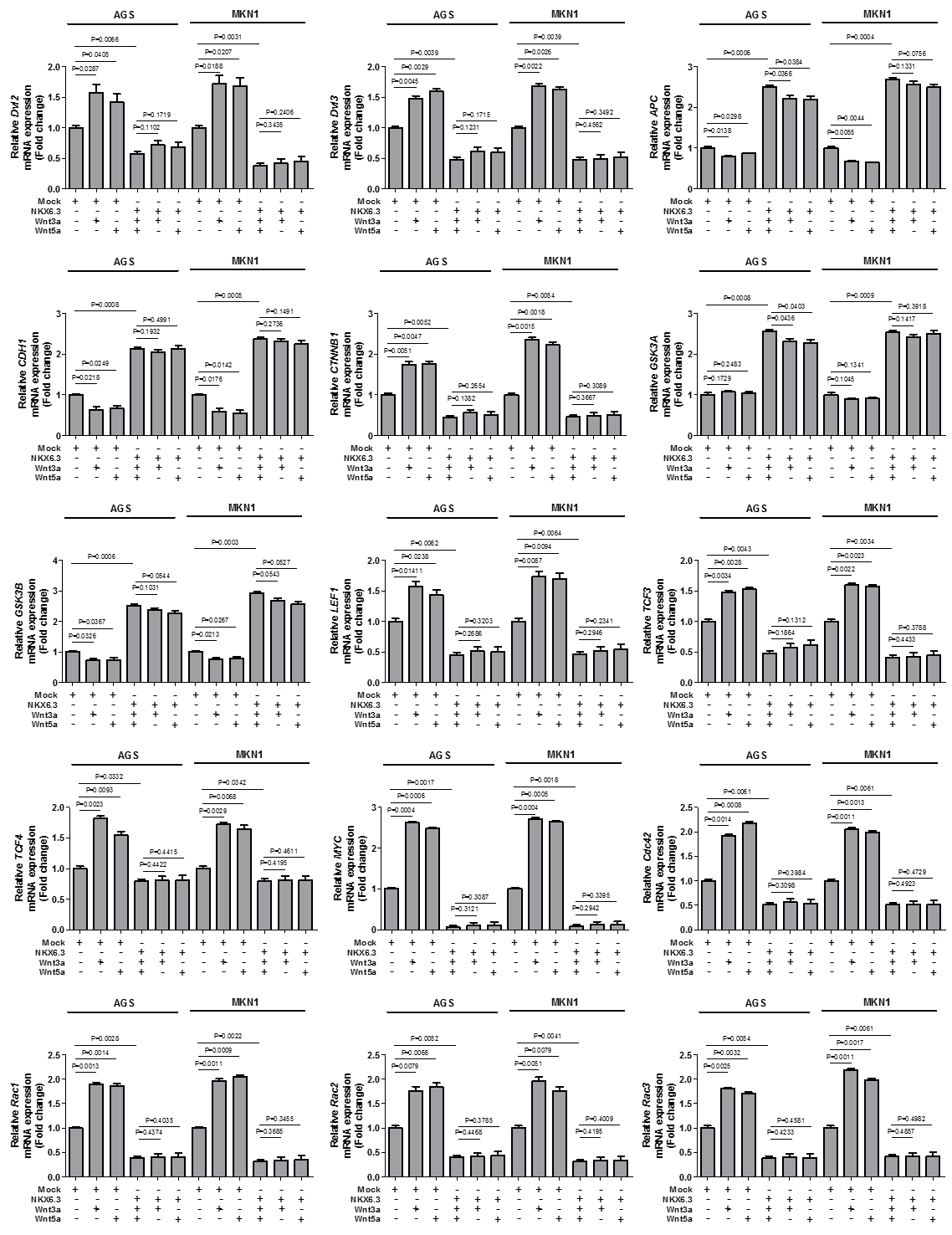
**

**
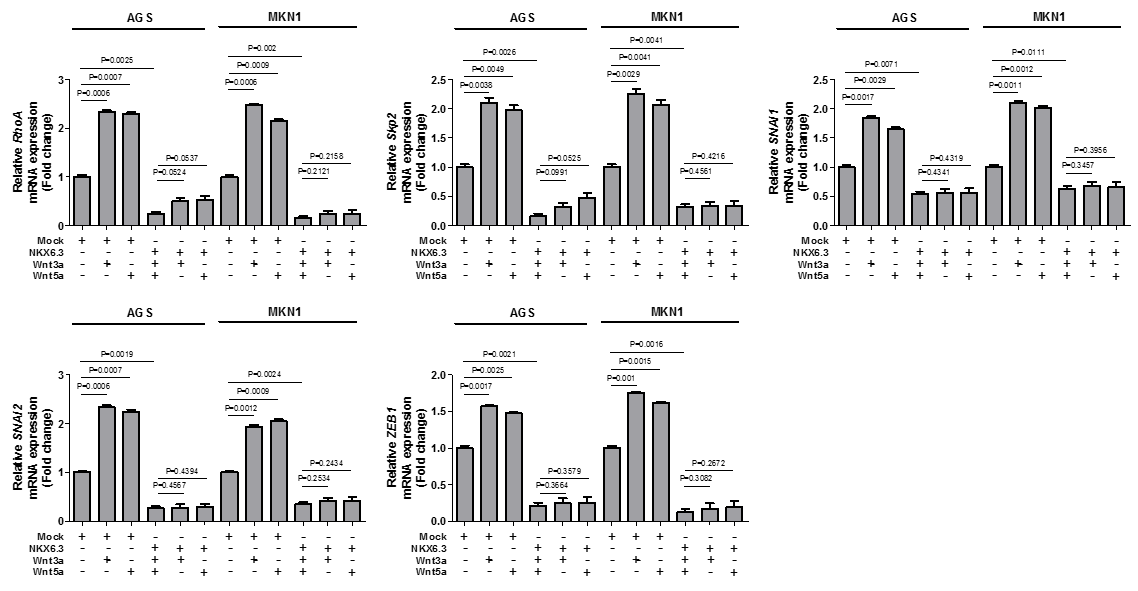
**

**Figure S4 (Related to Fig. 4c). Effects of NKX6.3 on Wnt-mediated gene expression.**

Wnt proteins induced changes in mRNA expression of Wnt/β-catenin- and Rho-GTPase signaling-related genes, such as *Dvl2*, *Dvl3*, *CTNNB1* and *RhoA*. NKX6.3 inhibited the Wnt-induced mRNA expression of these genes. The results are expressed as mean±SEM of three independent experiments. Data were statistically analyzed by Student's *t*-test.

**
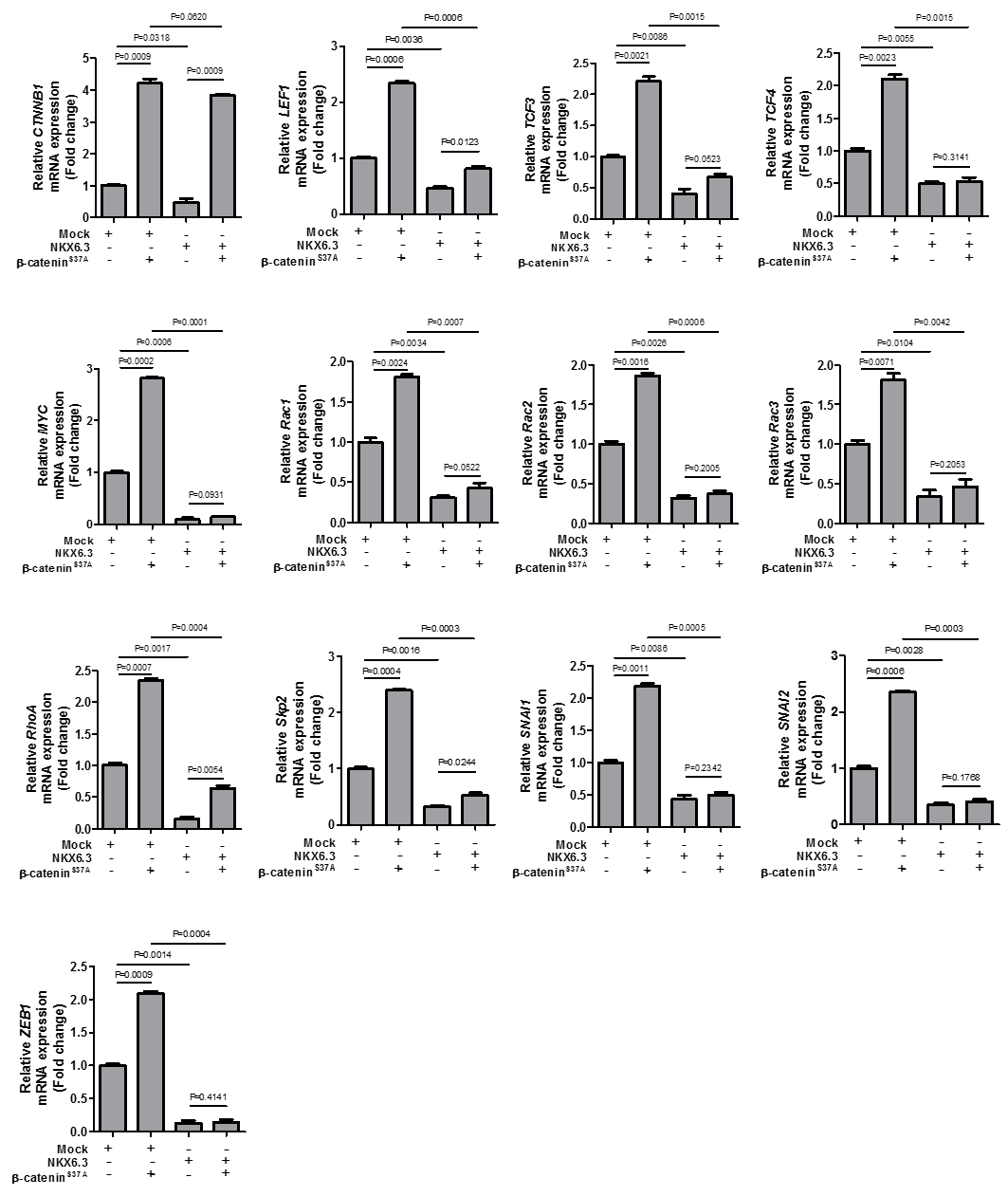
**

**Figure S5 (Related to Fig. 4d). Effects of NKX6.3 on mutant β-catenin-induced gene expression.**

Mutant β-catenin^S37A^ induced mRNA expression change including *c-Myc*, *Skp2* and *RhoA*. NKX6.3 significantly reduced the mRNA expression of the above genes. The results are expressed as mean±SEM of three independent experiments. Data were statistically analyzed by Student's *t*-test.

**
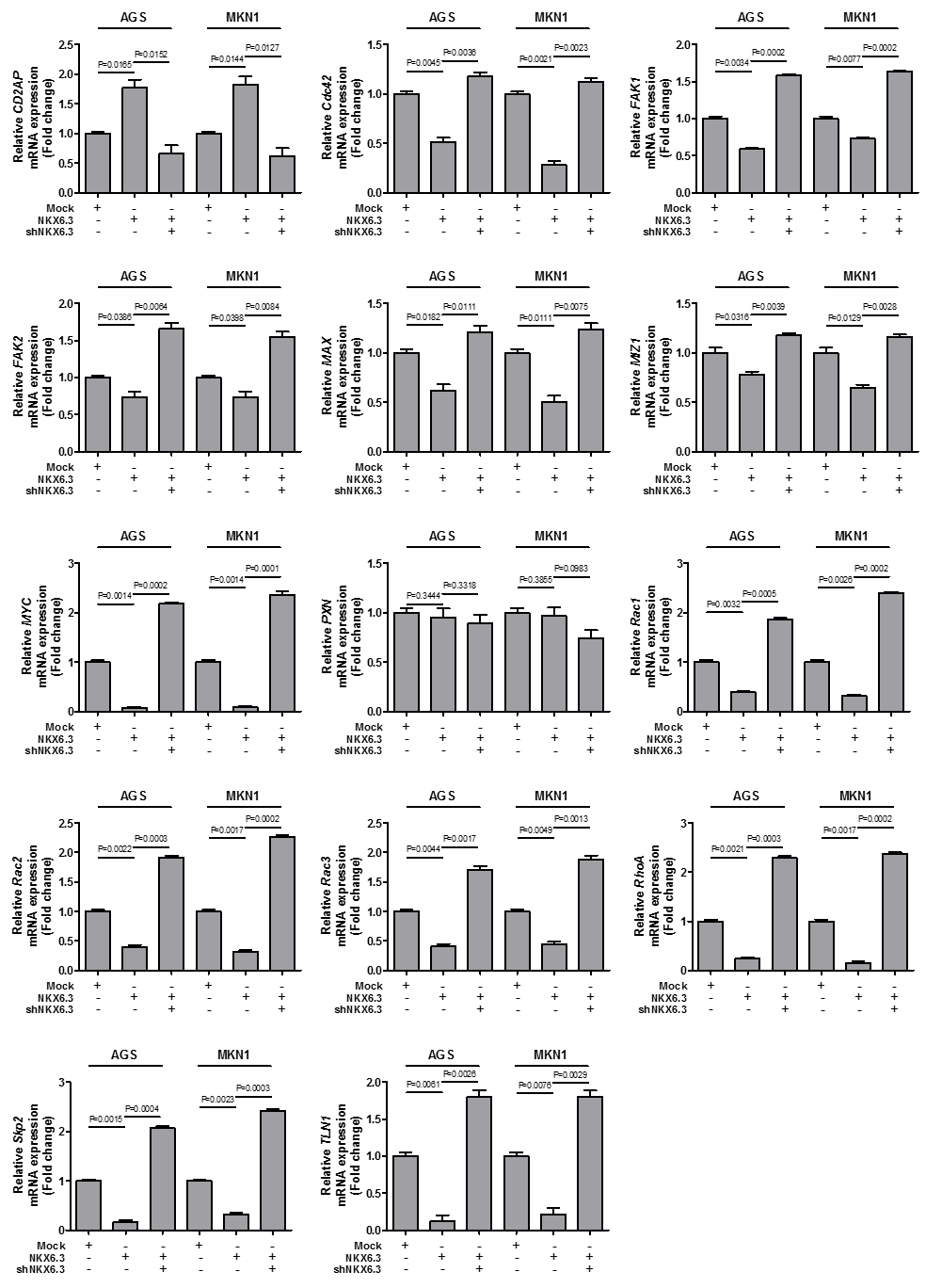
**

**Figure S6 (Related to Fig. 5g). Effects of NKX6.3 on Rho-GTPase family gene expression.**

mRNA expression of Rho-GTPase signaling pathway-related genes modulated by NKX6.3 in AGS and MKN1 cells using quantitative real-time PCR. NKX6.3 significantly reduced mRNA expression of Rho-GTPase family genes. The results are expressed as mean±SEM of three independent experiments. Data were statistically analyzed by Student's *t*-test.

**
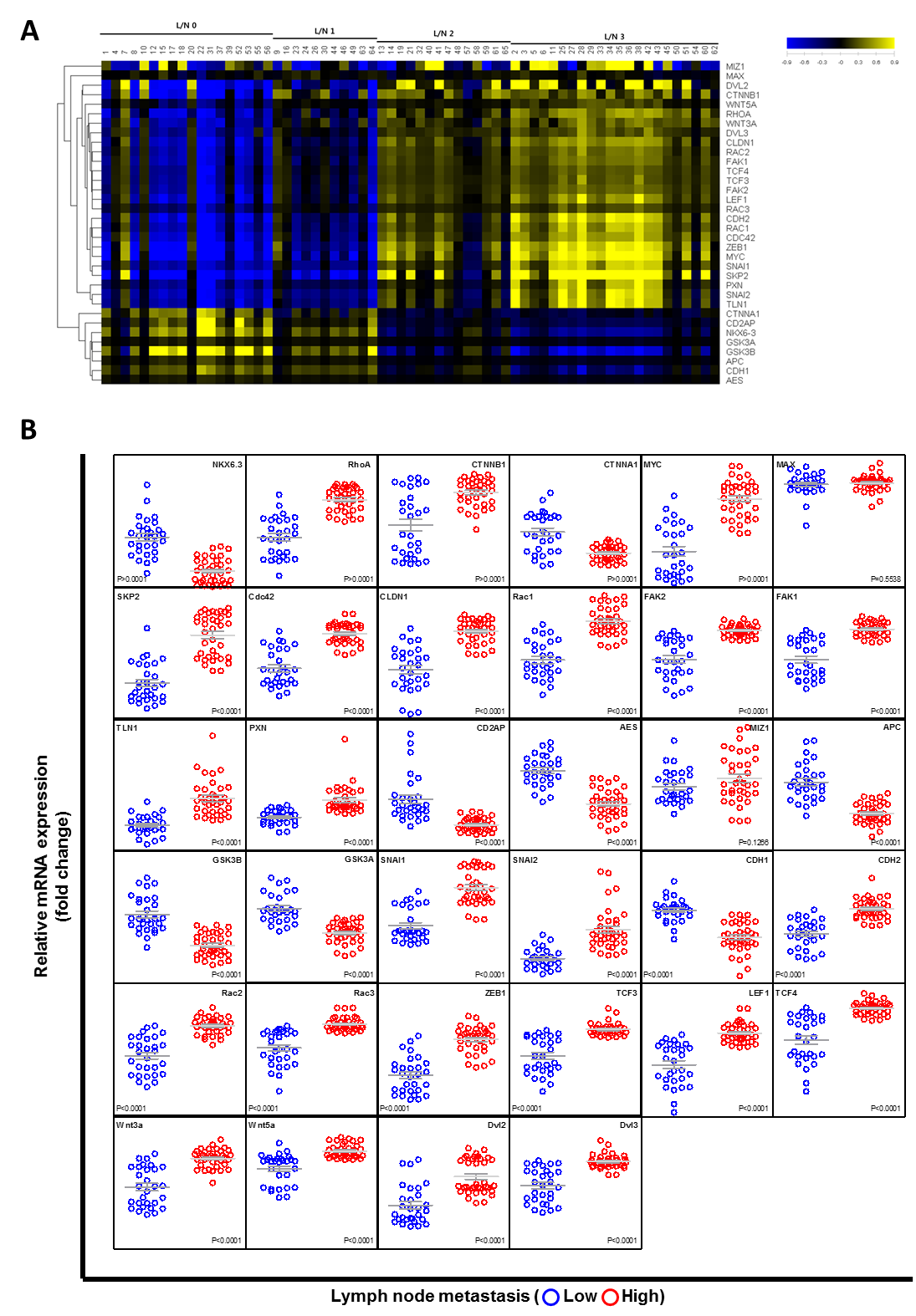
**

**
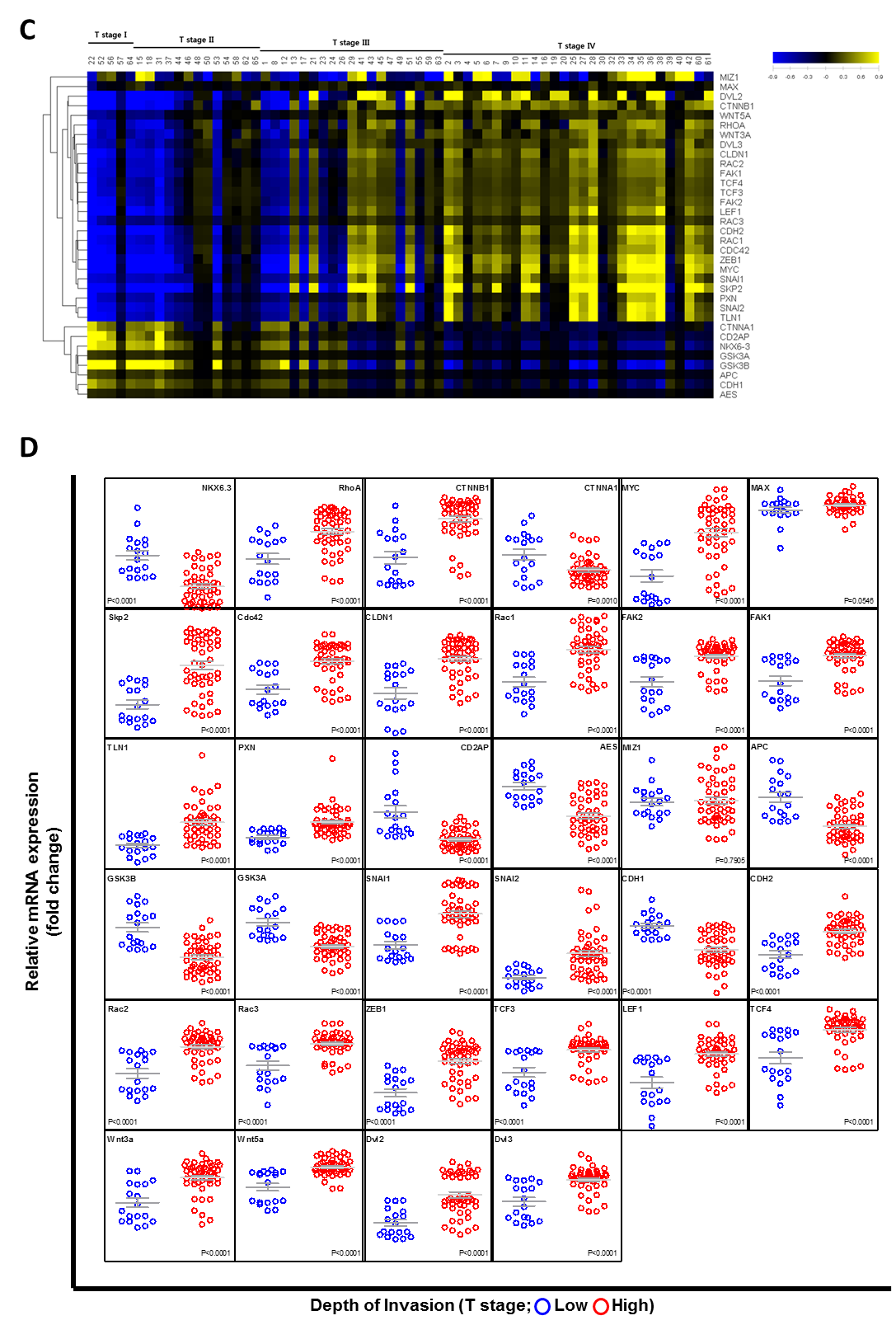
**

**Figure S7 (Related to Fig. 7). Expression patterns of NKX6.3 and Wnt/β-catenin, Rho-GTPase signaling-related genes in gastric cancer tissues according to L/N (lymph node) metastasis and T (tumor) stage.**

(A) Heat-maps demonstrate the expression ratios of NKX6.3, 33 Wnt/β-catenin and Rho-GTPase signaling-related genes, examined using quantitative real-time PCR in 65 gastric cancer tissues with lymph node metastasis. On the scale bar, yellow indicates up-regulation and blue indicates down-regulation of mRNA compared to non-tumorous gastric mucosal tissues. Data are expressed as medians of three independent experiments.

(B) Expression of NKX6.3 and 33 Wnt/β-catenin and Rho-GTPase signaling pathway genes in gastric cancer tissues with lymph node metastasis (blue dot, L/N 0, 1; red dot, L/N 2, 3). Data are expressed as medians from three independent experiments. Data were statistically analyzed by Student's *t*-test.

(C) Heat-maps demonstrate the expression ratios of NKX6.3, 33 Wnt/β-catenin and Rho-GTPase signaling-related genes, examined using quantitative real-time PCR in 65 gastric cancer tissues with depth of invasion (T stage). On the scale bar, yellow indicates up-regulation and blue indicates down-regulation of mRNA compared to non-tumourous gastric mucosal tissues. Data are expressed as medians of three independent experiments.

(D) Expression of NKX6.3 and 33 Wnt/β-catenin and Rho-GTPase signaling pathway genes in gastric cancer tissues with depth of invasion (blue dot, T 1, 2; red dot, T 3, 4). Data are expressed as medians from three independent experiments. Data were statistically analyzed by Student's *t*-test.


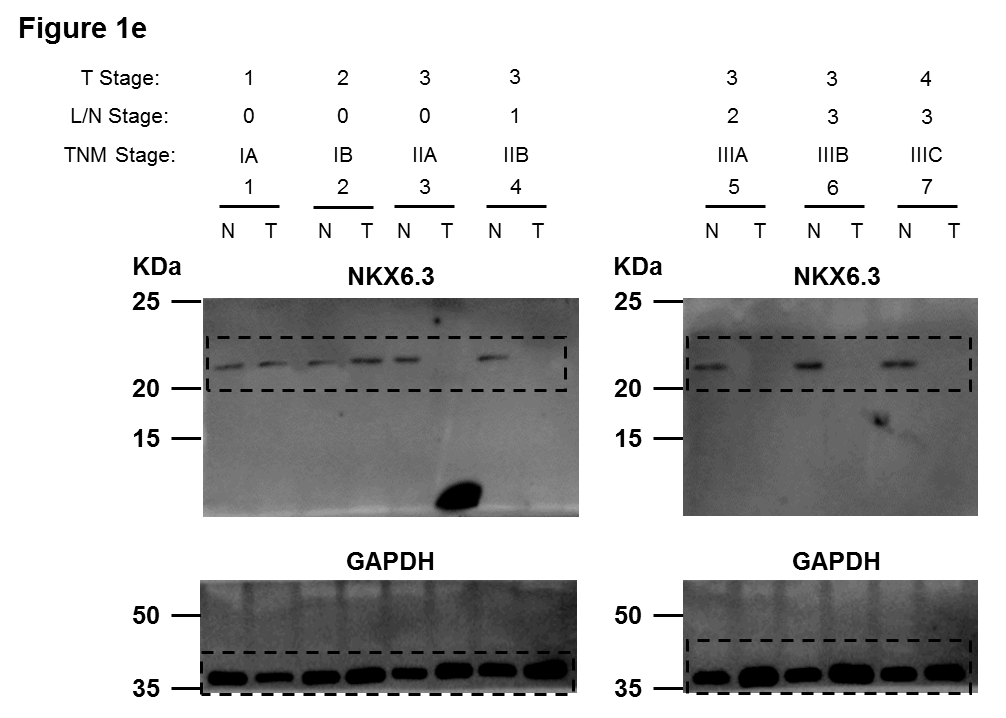


**Figure S8 (Related to Fig. 1e).** Unprocessed photographs of bigger sections of the Western blots with size markers corresponding to the indicated Western blots in the main figures. Black squares indicate cutting of Western blots as depicted in main figures. Western blots for detected proteins were run in parallel with Actin control blots with the same loading and running order.


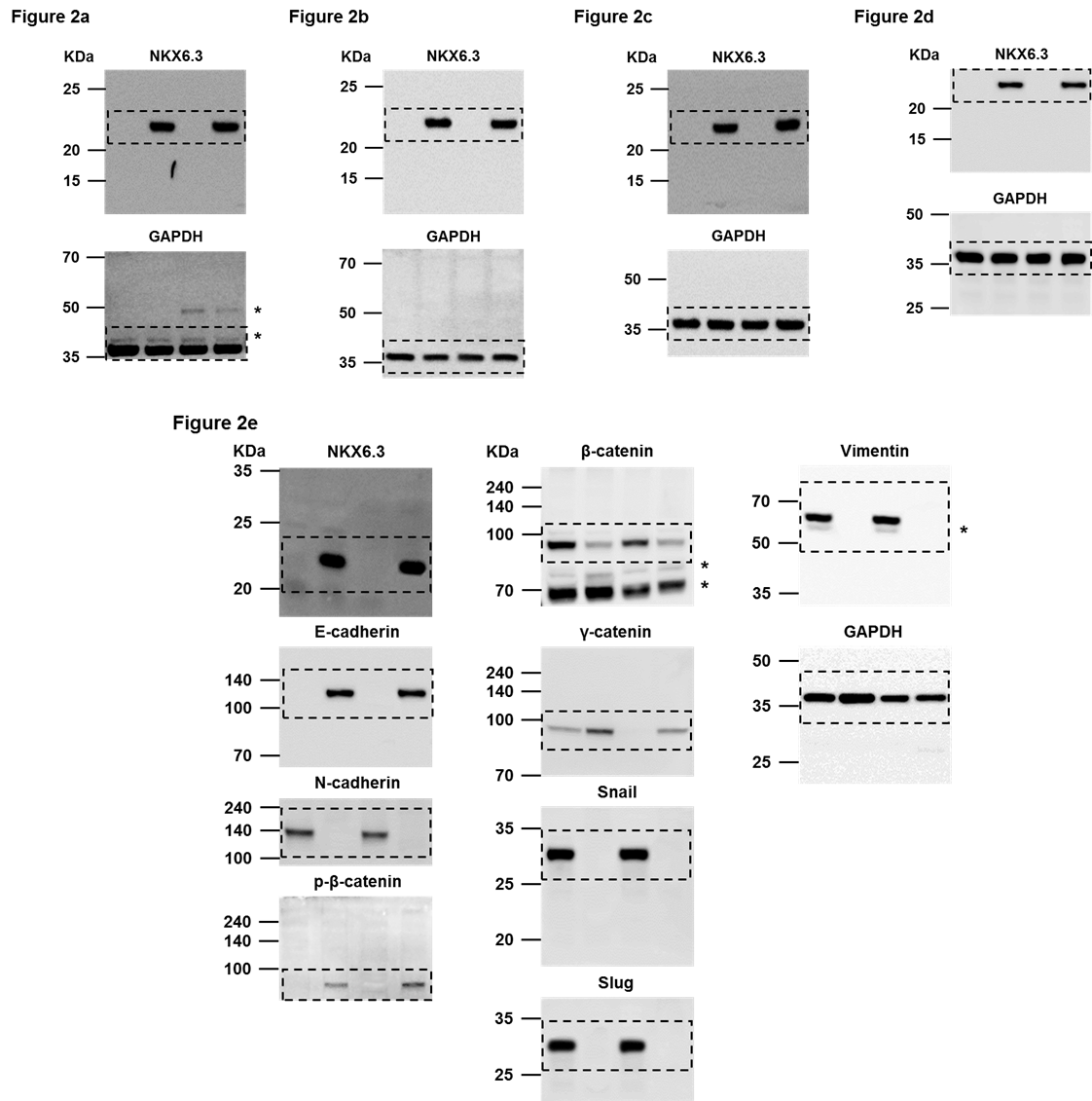


**Figure S9 (Related to Fig. 2a-e).** Unprocessed photographs of bigger sections of the Western blots with size markers corresponding to the indicated Western blots in the main figures. Black squares indicate cutting of Western blots as depicted in main figures. Western blots for detected proteins were run in parallel with Actin control blots with the same loading and running order.


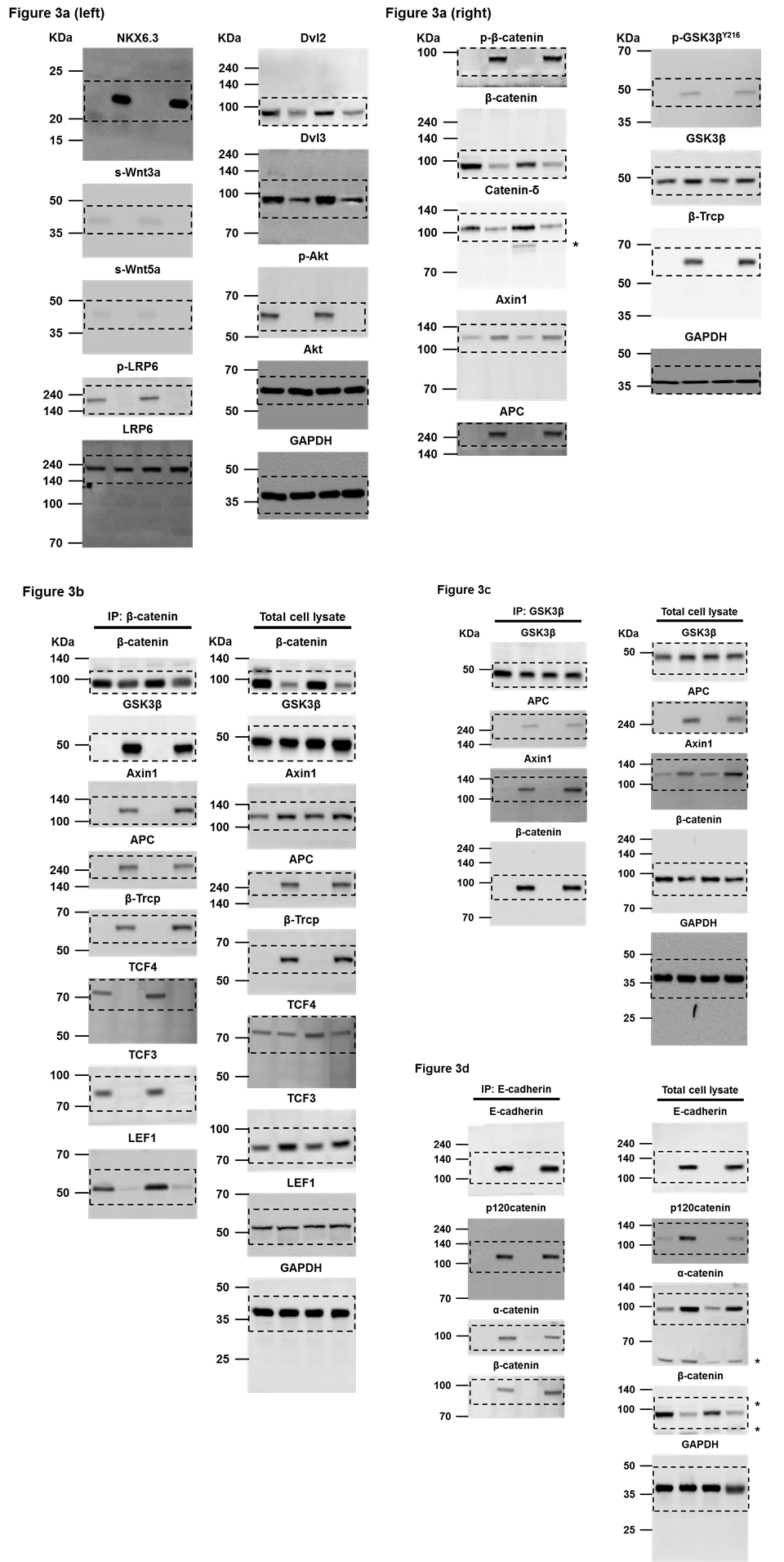


**Figure S10 (Related to Fig. 3a-d).** Unprocessed photographs of bigger sections of the Western blots with size markers corresponding to the indicated Western blots in the main figures. Black squares indicate cutting of Western blots as depicted in main figures. Western blots for detected proteins were run in parallel with Actin control blots with the same loading and running order.


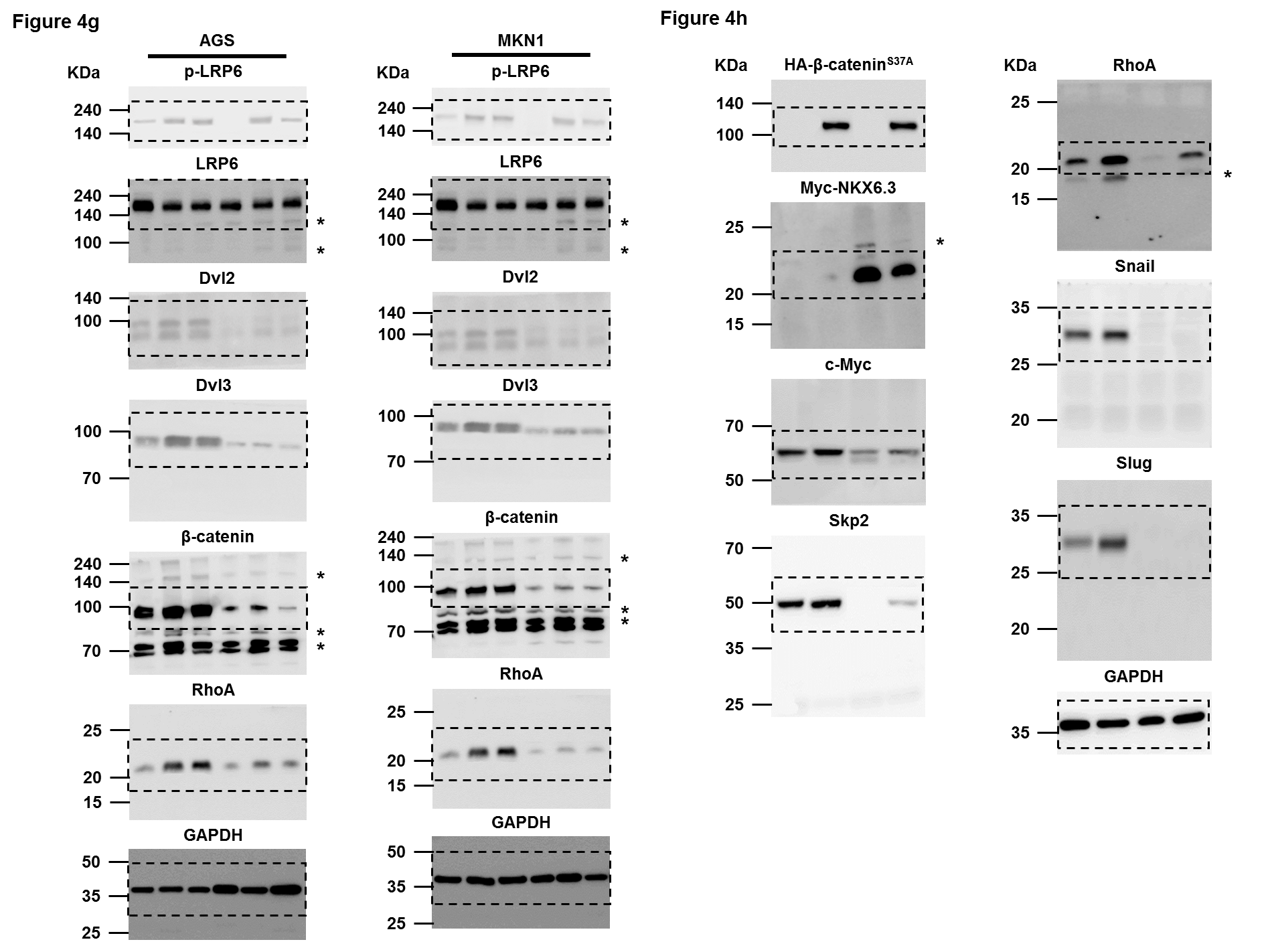


**Figure S11 (Related to Fig. 4g and h).** Unprocessed photographs of bigger sections of the Western blots with size markers corresponding to the indicated Western blots in the main figures. Black squares indicate cutting of Western blots as depicted in main figures. Western blots for detected proteins were run in parallel with Actin control blots with the same loading and running order.


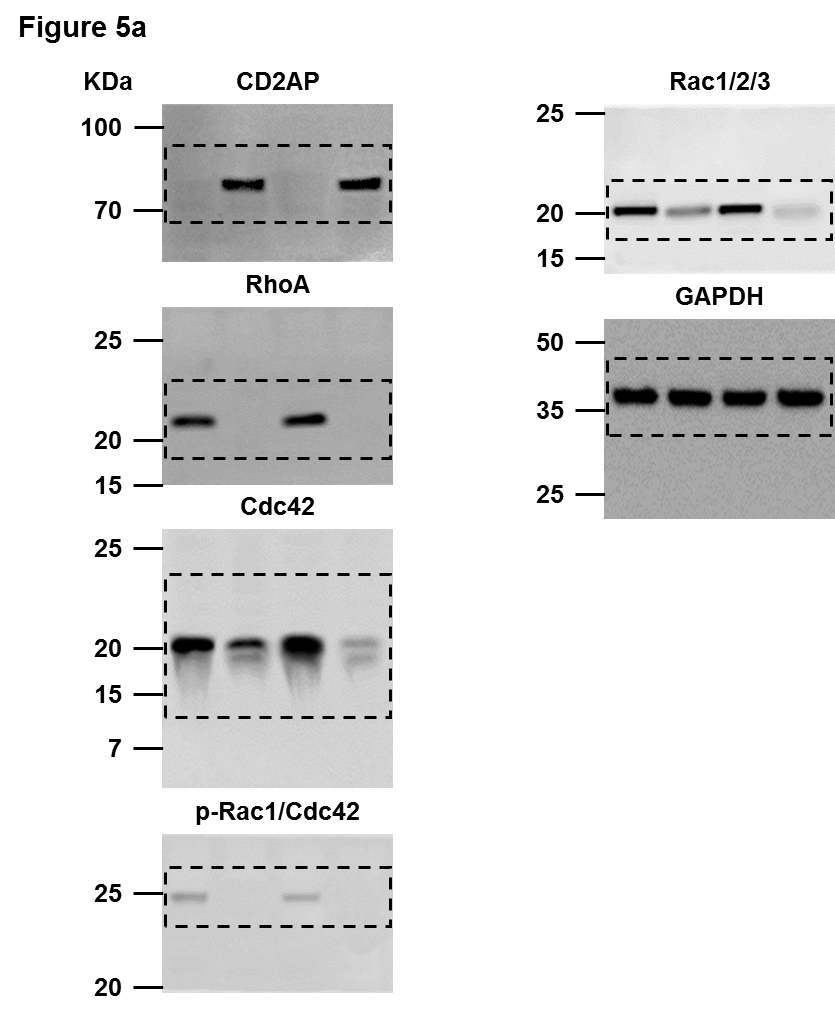


**Figure S12 (Related to Fig. 5a).** Unprocessed photographs of bigger sections of the Western blots with size markers corresponding to the indicated Western blots in the main figures. Black squares indicate cutting of Western blots as depicted in main figures. Western blots for detected proteins were run in parallel with Actin control blots with the same loading and running order.


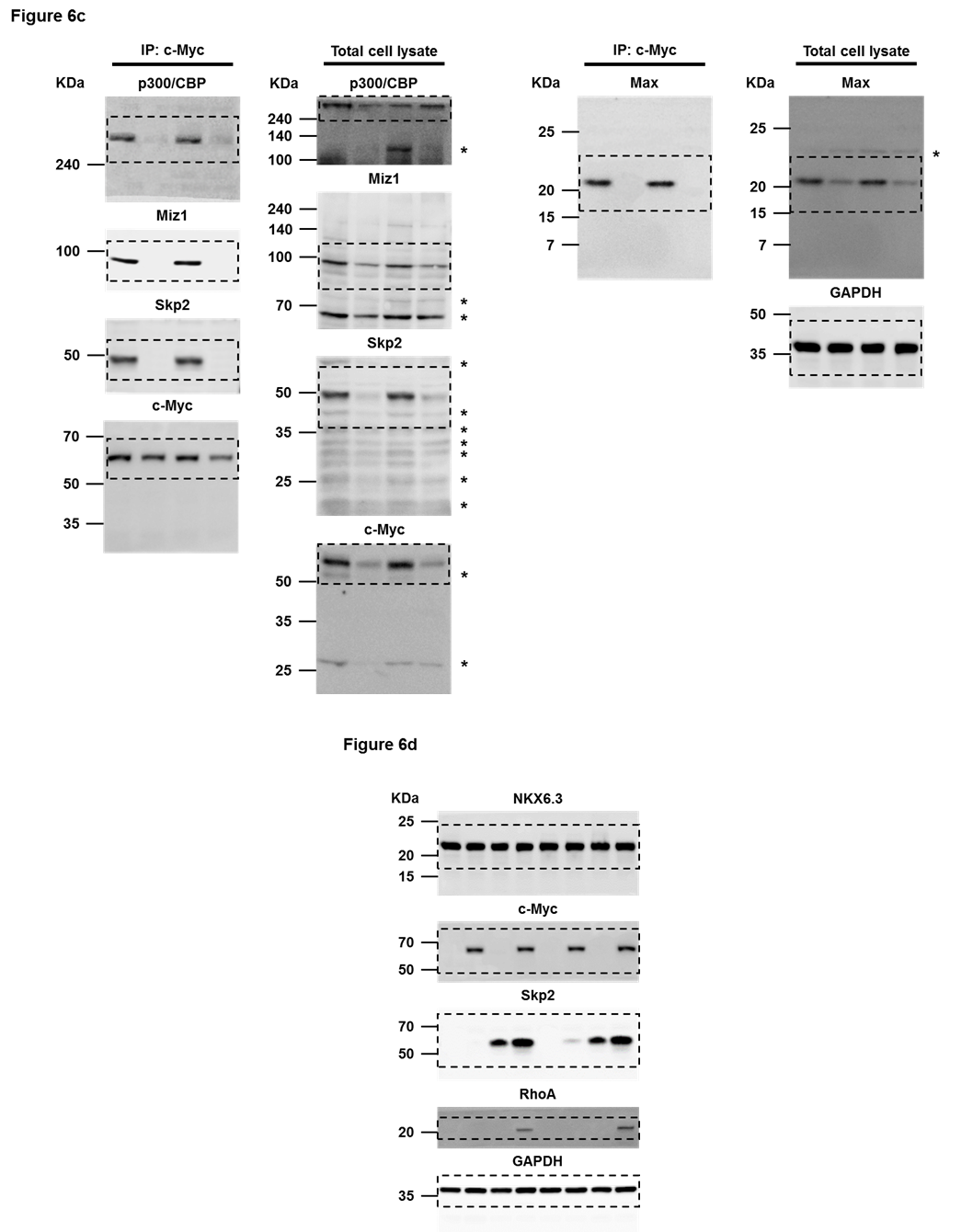


**Figure S13 (Related to Fig. 6c and d).** Unprocessed photographs of bigger sections of the Western blots with size markers corresponding to the indicated Western blots in the main figures. Black squares indicate cutting of Western blots as depicted in main figures. Western blots for detected proteins were run in parallel with Actin control blots with the same loading and running order.


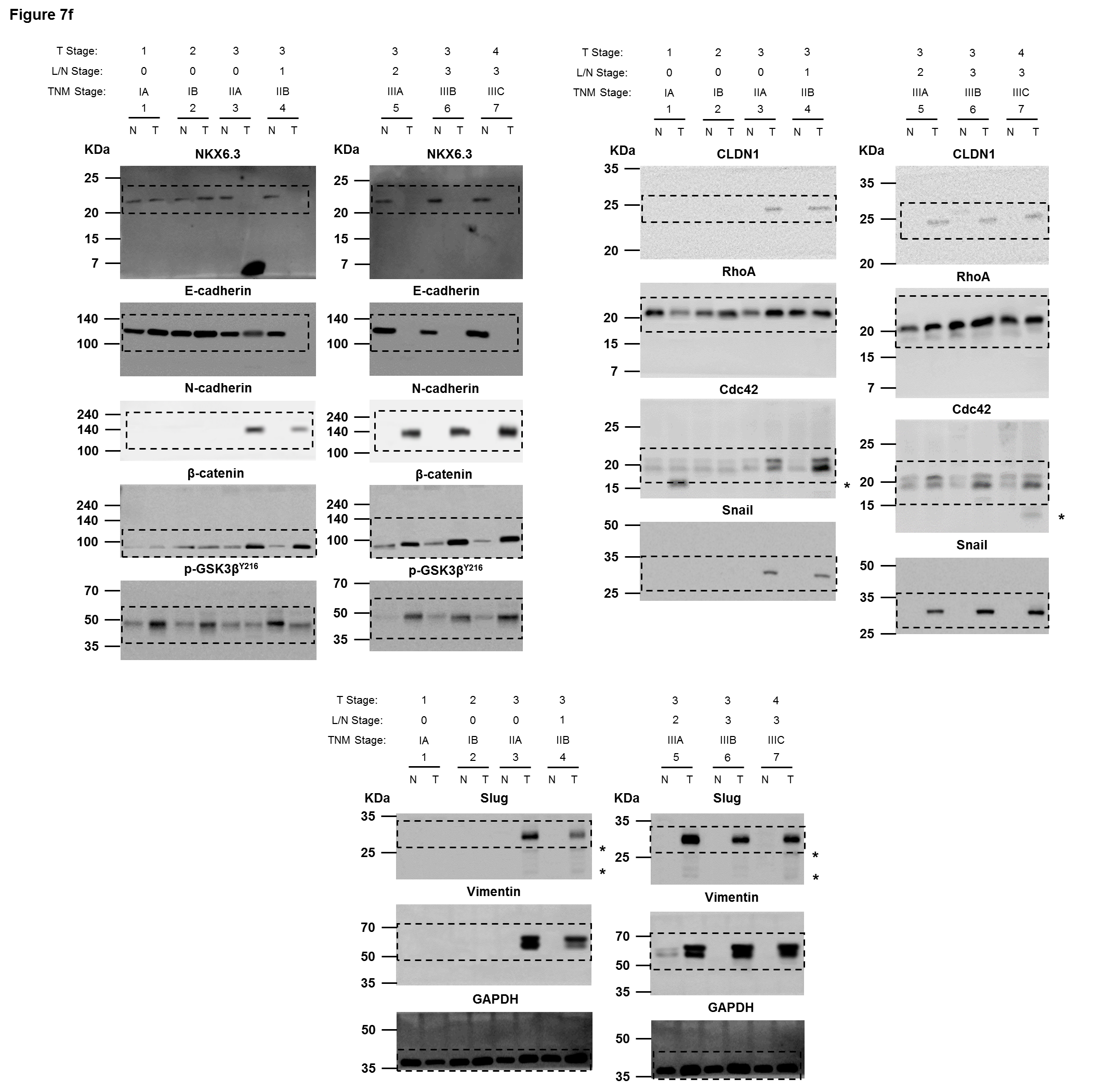


**Figure S14 (Related to Fig. 7f).** Unprocessed photographs of bigger sections of the Western blots with size markers corresponding to the indicated Western blots in the main figures. Black squares indicate cutting of Western blots as depicted in main figures. Western blots for detected proteins were run in parallel with Actin control blots with the same loading and running order.

**Supplemental Table S1. Association between NKX6.3 expression and clinicopathologic parameters.**

|  | NKX6.3 mRNA expression  (fold changes) | P value | Western blot | | P value | Immunohistochemistry | | P value |
| --- | --- | --- | --- | --- | --- | --- | --- | --- |
|  |  |  | NKX6.3 expression | |  | NKX6.3 expression | |  |
|  |  |  | **+** | **-** |  | + | - |  |
| Total | 0.5979±0.437351 |  | 13 | 52 |  | 33 | 124 |  |
| Age |  | 0.2608 |  |  | 0.9478 |  |  | 0.6063 |
| <60 | 0.541334±0.407449 |  | 5 | 17 |  | 15 | 65 |  |
| >60 | 0.631006±0.453089 |  | 8 | 35 |  | 18 | 59 |  |
| Gender |  | 0.3383 |  |  | 0.7129 |  |  | 0.1341 |
| Male | 0.618096±0.462869 |  | 11 | 39 |  | 25 | 74 |  |
| Female | 0.530565±0.336679 |  | 2 | 13 |  | 8 | 50 |  |
| Site |  | 0.0965 |  |  | 0.2197 |  |  | 0.209 |
| Upper | 0.431938±0.285488 |  | 0 | 8 |  | 6 | 10 |  |
| Middle | 0.687337±0.444373 |  | 7 | 18 |  | 10 | 48 |  |
| Lower | 0.569511±0.452224 |  | 6 | 26 |  | 17 | 66 |  |
| Lauren`s |  | 0.0025 |  |  | 0.2819 |  |  | 0.7539 |
| Intestinal | 0.457584±0.404995 |  | 10 | 29 |  | 13 | 55 |  |
| Diffuse | 0.691439±0.435491 |  | 3 | 23 |  | 20 | 69 |  |
| L/N^a^ |  | <0.0001 |  |  | <0.0001 |  |  | 0.0001 |
| N0 | 1.028411±0.414316 |  | 12 | 6 |  | 2 | 1 |  |
| N1 | 0.826163±0.226375 |  | 1 | 10 |  | 9 | 11 |  |
| N2 | 0.475026±0.192338 |  | 0 | 14 |  | 12 | 22 |  |
| N3 | 0.209715±0.200001 |  | 0 | 22 |  | 9 | 91 |  |
| Depth of invasion |  | <0.0001 |  |  | 0.0001 |  |  | 0.0001 |
|  |  |  |  |  |  |  |  |  |
| T1 | 1.327406±0.40888 |  | 4 | 1 |  | 7 | 1 |  |
| T2 | 0.9147893±0.320785 |  | 5 | 8 |  | 9 | 30 |  |
| T3 | 0.641084±0.342913 |  | 4 | 15 |  | 17 | 90 |  |
| T4 | 0.291144±0.232447 |  | 0 | 28 |  | 0 | 3 |  |
| Stage^b^ |  | <0.0001 |  |  | <0.0001 |  |  | 0.0001 |
| I | 1.344463±0.293249 |  | 9 | 0 |  | 2 | 0 |  |
| II | 0.869394±0.163808 |  | 4 | 12 |  | 17 | 12 |  |
| III | 0.329112±0.218631 |  | 0 | 39 |  | 14 | 111 |  |
| IV | 0.017445±0.004887 |  | 0 | 1 |  | 0 | 1 |  |

^a^Lymph node metastasis, ^b^TNM stage

**Supplemental Materials and Methods**

**Cell culture and establishment of NKX6.3 stable cell lines.**

AGS cells were originally derived from gastric adenocarcinoma, MKN1 cells were originally derived from gastric adenosquamous carcinoma. Both cell lines were obtained from the American Type Culture Collection. Both cells were cultured at 37^o^C in 5% CO_2_ in RPMI-1640 medium (Gibco, Auckland, New Zealand) supplemented with 10% fetal bovine serum (FBS; Biowest, Miami, USA), 1% penicillin/streptomycin (Invitrogen, CA, USA). Complete *NKX6.3*-cDNA was cloned into the expression vector pCMV6-Myc-DDK (Origene). AGS and MKN1 cells were transiently transfected with expression plasmids (5 μg total DNA) in 60 mm-diameter dishes using Lipofectamine Plus transfection reagent (Invitrogen), according to the manufacturer's recommendations. To obtain a stable cell line, selection pressure was maintained by supplementing the cultures with G418 (500 μg/ml; Gibco) for a period of 4-8 weeks. Clonal populations of cells from each well and transferring them 6-well plate. Stable expression of NKX6.3 was confirmed in AGS and MKN1 cells by western blot analysis.

**Chromatin immunoprecipitation (ChIP).**

ChIP assays were performed using the Thermo Scientific Pierce Agarose ChIP kit (Thermo Scientific Pierce), according to the manufacturer`s instructions. Briefly, cells were cross-linked with 1% formaldehyde and collected into lysis buffer (1% SDS, 10 mM EDTA, 50 mM Tris-HCl, pH 8.0, 1× protease inhibitor mixture). Cell lysates were digested with micrococcal nuclease, followed by immunoprecipitating with rabbit Myc-DDK or c-Myc antibody. Immunoprecipitation with a normal rabbit IgG (Thermo Scientific) was used as a negative control. After incubation with the protein A/G Plus agarose resin, immunoprecipitates were washed and then heated at 65 °C for 1.5 h to reverse the formaldehyde cross-linking. DNA fragments were purified with the DNA clean-up column and reagents included in the Pierce agarose ChIP kit.

The primer used are listed in the table below:

| Gene | Primer |
| --- | --- |
| *APC* | F: CCTTGGGCAAGGGACTTAAT  R: GGAGAGAAGGAAGGCTTCAA |
| *CDH1* | F: AGTGAAGTGGCTCCAGTGCT  R: GGCTAGTTGGGAGGTGAGAA |
| *CTNNB1* | F: TTGATAATGTATTTTTCTTTGATTTAATG  R: TACATTTTATTAAAGAAAATGTAAAATTTGC |
| *Cdc42* | F: CCTCACAAAGTGCTGGGATTA  R: CTACCACACTATCAAGCAGGTTTA |
| *Rac1* | F: ACTTGGACAACTGTTCTCACTC  R: TGTGATCGAGCTACTGCATTC |
| *Rac2* | F: GCTGCATTTATAGATGAAACACTTG  R: CATTGGAACATCACATTGTACCC |
| *RhoA* for NKX6.3 | F: CCTTATAGTTACTGTGTAATTAGTGCCA  R: ACCAATACACTTTCTTTGAGGATGA |
| *RhoA* for MYC | F: CTTCGCGTGCGTGAAGAGTTG  R: CATCCACTATTGCTCAGGAGC |
| *SNAI2* | F: TAGTGTCTTGGTGTCCAAAATCTAATC  R: TTAATGAAAGTAAAACATTTTAAACATGAAA |
| *Skp2* | F: GAGAATAATCTGTAAAGTCCTTCCT  R: TCTTACTAGGTTGTGAGGTCTAAT |
| *MYC* | F: ATTGTGCCACTGCACTCC  R: TGGGTATTTGGTTTGGCCTATT |
| *VIM* | F: GTATGTGGTCAGGTCATGCTAC  R: GCCCAAAGACCTGGATGTTTA |
| *Wnt3A* | F: GAGGAGCTGGGCAAAGAAT  R: TCTAACCTGGGTGACAGAGT |
| *Wnt5A* | F: GGGCATAATCTCAGCACACTAA  R: TCTAGTCCCAAGGACTTCAGAT |

The expression of gene promoter and enhancer were quantified by SYBR green-based real-time quantitative PCR (q-PCR).

**RNA isolation and quantitative reverse-transcriptase PCR.**

Total RNA was extracted from gastric cancer tissues and cell lines following the TRIzol Reagent (Invitrogen) protocol. RNA quality and concentration were assessed with a NanoDrop ND-1000 spectrophotometer. Two micrograms of total RNA was used in reverse transcription following the Superscript III (Invitrogen) protocol. For quantitative RT PCR, 50 ng of reverse transcribed cDNAs were amplified with the IQ5 optical system (Bio-rad) using SYBR Green Q-PCR Master Mix (Bio-rad). Primers for SYBR Green analysis were designed based on gene-specific non-homologous DNA sequences. The standard curve method was used for quantification of the relative amounts of gene expression products. This method provides unit-less normalized expression values that can be used for direct comparison of the relative amounts of target mRNA in different samples. All samples were tested in duplicate, and average values were used for quantification.

The sense and antisense primers of the genes are listed in the table below:

| Gene | Primer |
| --- | --- |
| *NKX6.3* | F: TCTTTCTGCTTCTGGGGTGT  R: GTCCAGCGGCTTGTTGTACT |
| *APC* | F: TTCCATAAGAACGGAGGGACA  R: TTTGACCGCAGTTTTTACTCCAGGGAAAATTACT |
| *CDH1* | F: AGTGAAGTGGCTCCAGTGCT  R: GGCTAGTTGGGAGGTGAGAA |
| *CTNNA1* | F: GCGGGGAGCTTGTTGTCT  R: AAGTTCTTGGCTGCCTGGAT |
| *CTNNB1* | F: AAAATGGCAGTGCGTTTAG  R: TTTGAAGGCAGTCTGTCGTA |
| *CD2AP* | F: GGCATGGGAATGTAGCAAGT  R: GTGGATGTGGCTGAATTCCT |
| *Cdc42* | F: GATGGTGCTGTTGGTAAA  R: TAACTCAGCGGTCGTAAT |
| *CLND1* | F: CTGCCCCAGTGGAGGATTTA  R: CATGGCCTGGGCGGT |
| *Rac1* | F: AAGCTGACTCCCATCACCTATCCG  R: CGAGGGGCTGAGACATTTACAACA |
| *Rac2* | F: AAGAAGCTGGCTCCCATCACCTAC  R: AACACGGTTTTCAGGCCTCTCTG |
| *RhoA* | F: CAGAAAAGTGGACCCCAGAA  R: GCAGCTGCTCTCGTAGCCATTTC |
| *Slug* | F: TGTTGCAGTGAGGGCAAGAA  R: GACCCTGGTTGCTTCAAGGA |
| *Skp2* | F: CTGTCTCAAGGGGTGATTGC  R: TTCGATAGGTCCATGTGCTG |
| *MYC* | F: AATGAAAAGGCCCCCAAGGTAGTTATCC  R: GTCGTTTCCGCAACAAGTCCTCTTC |
| *Vimentin* | F: TGTCCAAATCGATGTGGATGTTTC  R: TTGTACCATTCTTCTGCCTCCTG |
| *Wnt3A* | F: CCTGCACTCCATCCAGCTACA  R: GACCTCTCTTCCTACCTTTCCCTTA |
| *Wnt5A* | F: GAAATGCGTGTTGGGTTGAA  R: ATGCCCTCTCCACAAAGTGAA |
| *Dvl2* | F: TGAGCAACGATGACGCTGTG  R: GCAGGGTCAATTGGCTGGA |
| *Dvl3* | F: ACAATGCCAAGCTACCATGCTTC  R: AGCTCCGATGGGTTATCAGCAC |
| *GSK3B* | F: TCGAGAGCTCCAGATCATGAGAA  R: CGGAACATAGTCCAGCACCAGA |
| *TCF4* | F: CTGCCTTAGGGACGGACAAAG  R: TGCCAAAGAAGTTGGTCCATTTT |
| *LEF1* | F: AATGAGAGCGAATGTCGTTGC  R: GCTGTCTTTCTTTCCGTGCTA |
| *TCF3* | F: GTACCCCTTCCTGATGATCC  R: GACCTCGTGTCCTTGACTG |
| *Miz1* | F: GTGGTGGACGGTGTTCACTT  R: GCCACGGCCAGCACATCAT |
| *Max* | F: GAACGAAAACGTAGGGACCA  R: TGCTGGTGTGTGTGGTTTTT |
| *Snail* | F: ACCACTATGCCGCGCTCTT  R: GGTCGTAGGGCTGCTGGAA |
| *Rac3* | F: AAGAAGCTGGCACCCATCACCTAC  R: ATCGCCTCGTCAAACACTGTCTTC |
| *GSK3A* | F: GAGGGGAGCCCAATGTCTC  R: GATTTGTTCCCGGGTTGGTG |
| *CDH2* | F: GGCATAGTCTATGGAGAAGT  R: GCTGTTGTCAGAAGTCTCTC |
| *ZEB1* | F: GCACCTGAAGAGGACCAGAG  R: TGCATCTGGTGTTCCATTTT |
| *FAK1* | F: TTATTGGCCACTGTGGATGA  R: TACTCTTGCTGGAGGCTGGT |
| *FAK2* | F: AAGTTCATGAGCGAGGCAGT  R: GAGTTCTTGTTCCGCTCCAG |
| *TLN1* | F: CCCTGATGTGCGGCTTCG  R: TGTCCTGTCAACTGCTGCTTC |
| *PXN* | F: CCCTGACGAAAGAGAAGCCTAAG  R: AGATGCGTGTCTGCTGTTGG |
| *AES* | F: TGTCCTACGGATTGAACATCGA  R: GGCGCAAATCCCATTCAG |
| *β-actin* | F: GTT GCT ATC CAG GCT GTG  R: TGA TCT TGA TCT TCA TTG TG |

**Immunoblot and Immunofluorescence (IF).**

The effect of NKX6.3 on expression of Wnt/β-catenin, Rho-GTPase signaling and EMT-related proteins including E-cadherin, β-catenin, γ-catenin, Snail, Slug, vimentin, Zo-1, and ZEB1 was determined in AGS^Mock^, MKN1^Mock^, AGS^NKX6.3^ and MKN1^NKX6.3^ by Western blot, immunofluorescence and confocal microscopy. Briefly, for immunofluorescence assays, AGS^Mock^, MKN1^Mock^, AGS^NKX6.3^ and MKN1^NKX6.3^ cells were grown on 4-well chamber slides and fixed with 4% paraformaldehyde in 0.1 M phosphate buffer (pH 7.4), for 10 min at room temperature, rinsed in PBS, and incubated in 10% normal donkey serum for 30 min to block nonspecific binding. The cells were then incubated with a primary antibody in PBS containing 0.5% Triton X-100 overnight. The cells were rinsed with PBS, and incubated with a Cy3-conjugated donkey anti-mouse or anti-rabbit IgG (1:200 dilution; Jackson Immunoresearch, West Grove, PA, USA) for 1 hr at room temperature. Counterstaining of cell nuclei was carried out by incubating the cells with DAPI (4',6-diamidino–2'-phenyindole; Roche; dilution 1:1000) for 10 min. Slides were viewed with a confocal microscope (LSM 510 Meta, Carl Zeiss Co., Ltd., Germany). Images were converted to TIFF format, and contrast levels were adjusted using Adobe Photoshop v. 7.0 (Adobe Systems, San Jose, CA, USA).

For western blot analysis, cell lysates were separated on a 10% polyacrylamide gel and transferred onto a Hybond PVDF membrane (Amersham Pharmacia Biotech, Piscataway, NJ, USA). After blocking, the membrane was subsequently probed with primary antibodies and then incubated with secondary antibodies. Protein bands were detected using enhanced chemiluminescence reagents (Amersham Pharmacia Biotech, Piscataway, NJ, USA).

The antibodies used are listed in the table below:

| Name | Company | Name | Company |
| --- | --- | --- | --- |
| NKX6.3  Myc-DDK taq  E-cadherin  N-cadherin  p-β-catenin^Ser33/37/Thr41^  β-catenin  γ-catenin  Snail  Slug  Vimentin  Wnt3a  Wnt5a  p-LRP6  LRP6  Dvl2  Dvl3  p-Akt  Akt  Catenin-δ  Axin1  APC | Atlas antibodies^a^  Santa cruz^b^  Cell signaling^c^  Cell signaling  Cell signaling  Cell signaling  Cell signaling  Abcam^d^  Abcam  Cell signaling  Cell signaling  Cell signaling  Cell signaling  Cell signaling  Cell signaling  Cell signaling  Cell signaling  Cell signaling  Cell signaling  Cell signaling  Cell signaling | p-GSK3β^Y218^  GSK3β  β-Trcp  TCF4  TCF3  LEF1  p120catenin  α-catenin  RhoA  HA-taq  c-Myc  Skp2  CD2AP  Cdc42  p-Rac1/Cdc42  Rac1/2/3  p300/CBP  Miz1  Max  Claudin1 | Cell signaling  Cell signaling  Santa cruz  Cell signaling  Cell signaling  Cell signaling  Cell signaling  Cell signaling  Cell signaling  Santa cruz  Cell signaling  Cell signaling  Cell signaling  Cell signaling  Cell signaling  Cell signaling  Cell signaling  Abcam  Abcam  Cell signaling |

^a^Atlas antibodies; Stockholm, Sweden, ^b^Santa Cruz biotechnology; Santa Cruz, CA, USA,

^c^Cell signaling Technology; Beverly, MA, USA, ^d^Abcam; Cambridge, MA, USA
